# Diagnostic value of cutaneous manifestation of SARS‐CoV‐2 infection*

**DOI:** 10.1111/bjd.19807

**Published:** 2021-03-02

**Authors:** A. Visconti, V. Bataille, N. Rossi, J. Kluk, R. Murphy, S. Puig, R. Nambi, R. C. E. Bowyer, B. Murray, A. Bournot, J. Wolf, S. Ourselin, C. J. Steves, T. D. Spector, M. Falchi

**Affiliations:** ^1^ Department of Twin Research & Genetic Epidemiology King’s College London London UK; ^2^ Dermatology Department West Herts NHS Trust Watford UK; ^3^ Zoe Global Limited London UK; ^4^ Dermatology Department Sheffield Teaching Hospitals NHS Foundation Trust Sheffield UK; ^5^ Dermatology Department Hospital Clinic of Barcelona University of Barcelona Barcelona Spain; ^6^ Institut d’Investigacions Biomèdiques August Pi I Sunyer Barcelona Spain; ^7^ University Hospitals of Derby and Burton NHS Foundation Trust Derby UK; ^8^ School of Biomedical Engineering & Imaging Sciences King’s College London London UK

## Abstract

**Background:**

One of the challenging aspects of SARS‐CoV‐2 infection is its diverse multisystemic disease presentation.

**Objectives:**

To evaluate the diagnostic value of cutaneous manifestations of SARS‐CoV‐2 infection and investigate their duration and timing in relation to other COVID‐19 symptoms.

**Methods:**

We used data from 336 847 UK users of the COVID Symptom Study app to assess the diagnostic value of body rash or an acral rash in SARS‐CoV‐2 infection, and data from an independent online survey of 11 544 respondents to investigate skin‐specific symptoms and collect their photographs.

**Results:**

Using data from the app, we show significant association between skin rashes and a positive swab test result (odds ratio 1·67, 95% confidence interval 1·42–1·97). Strikingly, among the respondents of the independent online survey, we found that 17% of SARS‐CoV‐2‐positive cases reported skin rashes as the first presentation, and 21% as the only clinical sign of COVID‐19. Together with the British Association of Dermatologists, we have compiled a catalogue of images of the most common skin manifestations of COVID‐19 from 400 individuals (https://covidskinsigns.com), which we have made publicly available to assist clinicians in recognition of this early clinical feature of COVID‐19.

**Conclusions:**

Skin rashes cluster with other COVID‐19 symptoms, are predictive of a positive swab test, and occur in a significant number of cases, either alone or before other classical symptoms. Recognizing rashes is important in identifying new and earlier cases of COVID‐19.

## Abstract 2

What is already known about this topic?

- Several studies conducted in hospital settings reported that patients with COVID‐19 presented with unusual skin rashes, including urticarial rashes, vesicular lesions and, less frequently, chilblains in fingers or toes.

What does this study add?

- We confirmed, in a community‐based setting that also includes milder forms of the disease, that the presence of a skin rash is predictive of SARS‐CoV‐2 infection.
- We provide a website with photos of skin manifestations to help healthcare professionals in diagnosing COVID‐19.
- Skin rashes should be taken into account to provide a quick COVID‐19 diagnosis to curb the spread of the disease.

**Linked Comment:** Naldi. *Br J Dermatol* 2021; **184**:793–794.

During the COVID‐19 pandemic, it became clear that the SARS‐CoV‐2 virus, while mainly targeting the lungs, also affected other organs.^1^ The first cases of COVID‐19 cutaneous manifestation were documented in China, but the prevalence was very low, at 0·2% in 1099 hospital cases.^2^ Italy then reported that 20% of patients on a COVID‐19 ward (*n* = 88) had skin clinical signs.^3^ Subsequently, other groups^4^, ^5^, ^6^, ^7^, ^8^, ^9^, ^10^ have described urticarial rashes, vesicular lesions, and less frequent cases of chilblains affecting fingers or toes (acral rash).

Here, using a population approach, we investigated the diagnostic value of body and acral rashes for SARS‐CoV‐2 infections using data from 336 847 users of the COVID Symptom Study app, and from an independent survey on COVID‐19‐related cutaneous symptoms in 11 544 respondents, 2 328 of whom also shared photographs of their skin complaints. The study is summarized in Figure 1.

**Figure 1:**
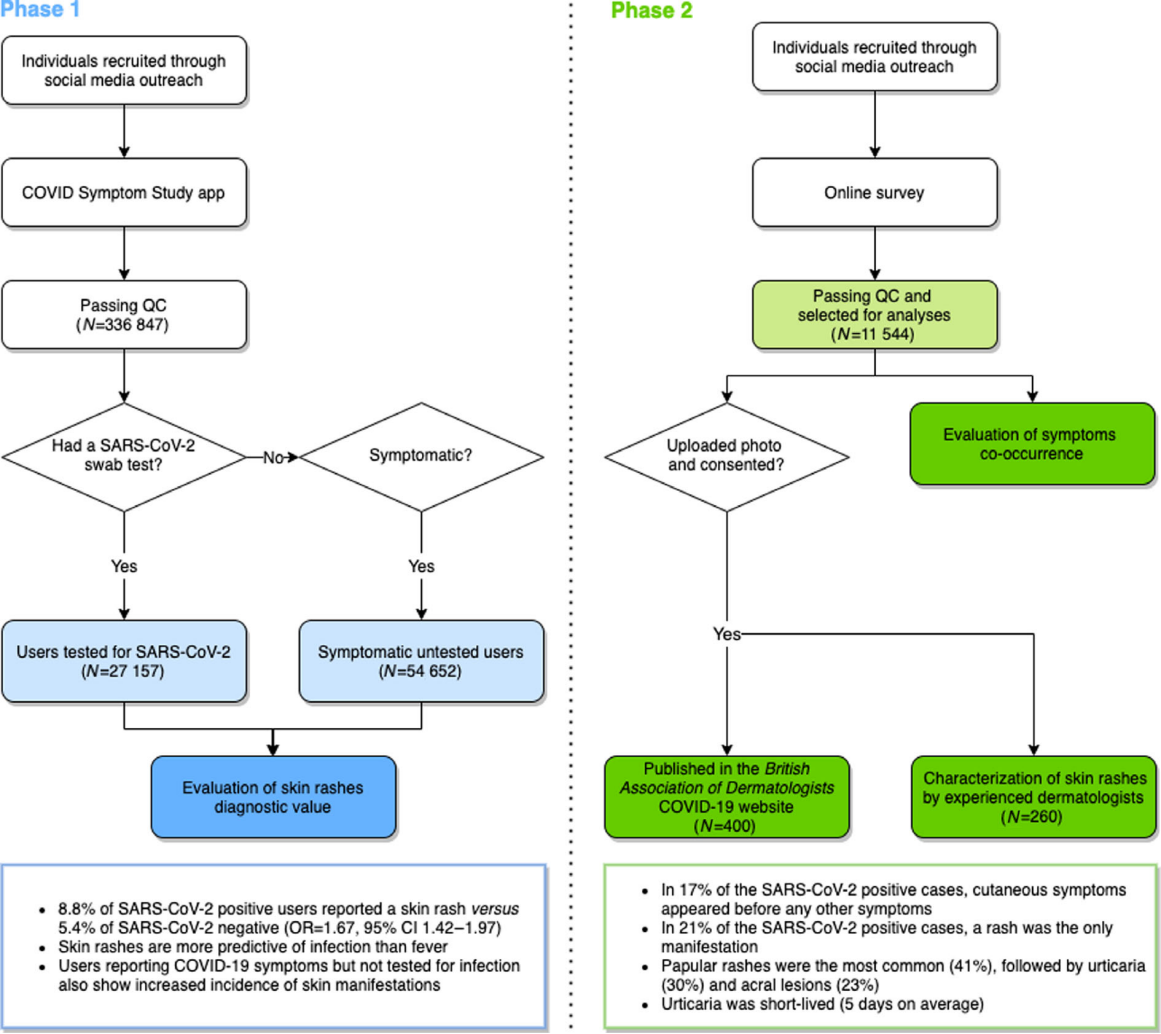
Study summary. We used data collected through the COVID Symptom Study app to investigate the ability of cutaneous symptoms to identify SARS‐CoV‐2 infection (Phase 1). An independent online survey was used to collect skin‐specific symptoms in order to explore their presentation, duration and timing in relation to other COVID‐19 symptoms, and to collect photographs of skin symptoms (Phase 2). Light‐coloured rectangles highlight the data used in the analyses; dark‐coloured rectangles represent the observations generated by this study; and the rectangles at the bottom summarize the main findings. CI, confidence interval; OR, odds ratio; QC, quality control.

## Materials and methods

### The COVID Symptom Study app

Users of the COVID Symptom Study app^11^ were recruited through social media outreach and included anyone able to download the app, either themselves or by proxy. On sign‐up the app collects data on sex, age and ethnicity (Asian, black, Chinese, Middle East, mixed or white); core health risk factors, including height, weight and common disease status (e.g. cancer, diabetes, and heart, kidney and lung disease); the use of medications (e.g. corticosteroids, immunosuppressants and blood pressure medications); and whether the user is a healthcare worker. From 7 May 2020, users were prompted to self‐report if they had ever had a SARS‐CoV‐2 test, including how it was performed (e.g. nose or throat swab, antibody testing) and the result (positive, negative, failed or waiting). Users could provide daily updates on their health status and the presence of up to 14 COVID‐19‐related symptoms: abdominal pain, chest pain, delirium, diarrhoea, fatigue, fever, headache, hoarse voice, anosmia, persistent cough, shortness of breath, skipped meals, sore throat and unusual muscle pains. From 29 April 2020, two cutaneous manifestations were also added: raised, red, itchy weals on the face or body or sudden swelling of the face or lips (body rash); and red/purple sores or blisters on the feet or toes (acral rash). Asking the participants to differentiate between a transient urticarial rash and a fixed erythematopapular or vesicular rash was problematic, so the body rashes were collected together, as done elsewhere.^4^

This study included residents in the UK, reporting a numerically plausible age range between 1 and 90 years, and who downloaded the app and entered regular data between 7 May and 22 June 2020, either themselves or via proxy. As the app does not perform any validation of user‐inputted data at the time of logging, we used the following criteria to exclude users reporting unreliable and extreme observations: for users aged 16 years or older, height, weight or body mass index (BMI) outside the ranges of 1·1–2·2 m, 40–200 kg and 15–55 kg m^−2^, respectively; and for users younger than 16 years old, height, weight or BMI outside two SDs from the sample’s mean for each age group. Pregnant women, and users who did not report their sex were also excluded.

Details on the protocol used for selecting the study sample are shown in Figure S1 (see Supporting Information). This resulted in 336 847 users, 17 407 of whom also provided valid (i.e. positive or negative) results for SARS‐CoV‐2 swab tests (hereafter: ‘tested users’). We further selected 54 652 symptomatic users (i.e. users reporting at least one of the 16 collected symptoms during their daily log history) who did not consider themselves to have been already infected when first registering with the app and were not tested via nose/throat swab (hereafter: ‘symptomatic untested users’). These were divided into two groups: (i) those reporting at least one of the three main symptoms of COVID‐19 (fever, persistent cough and/or anosmia) either at the time of logging or in retrospect, and who, according to the UK National Health Service (NHS) guidelines, would require isolation and testing; and (ii) those who did not (Figure S2; see Supporting Information).

### The skin rash survey

To collect more detailed information on the duration and timing of cutaneous symptoms with respect to other COVID‐19 symptoms, and to create a repository of photographs, we delivered an independent online questionnaire via SurveyMonkey (www.surveymonkey.co.uk), asking whether the rash was the only symptom, how many days it lasted, and, if other COVID‐19‐related symptoms were present, whether the rash started before, during or after them. The questionnaire was open from 12 to 17 June 2020, and 29 966 individuals participated. This survey was advertised on social media and was not specifically addressed to the app’s users. However, we cannot exclude that some of the surveyees were also users of the app, although we are unable to link individuals from the two study samples.

We removed 18 422 surveyees not reporting their sex, not giving a numerically plausible age (between 1 and 90 years), not reporting the duration of their cutaneous symptoms, or reporting symptoms lasting > 6 weeks (Figure S3; see Supporting Information). Of the 11 544 surveyees passing this quality control, 2 328 uploaded a photograph of their rash and gave consent for sharing. From these, we randomly selected 260 photographs from people of either sex, with either a positive SARS‐CoV‐2 test or reporting at least one of the three main symptoms included in the UK NHS guidelines (i.e. fever, persistent cough and/or anosmia). The 260 photographs were blindly assessed and independently categorized by four experienced dermatologists. Classifications were accepted when at least three dermatologists agreed on the diagnosis.

In collaboration with the British Association of Dermatologists, the complete photograph database was subsequently examined by four consultant dermatologists, and about 400 photographs were classified by consensus to create a large library of curated photographs divided into several types of rashes.

### Ethical statement

The study was approved by the King’s College London Research Ethics Committee, REMAS ID 18210, review reference LRS‐19/20‐18210. All app users and surveyees provided informed consent, either themselves or by proxy. Additional consent was sought for the sharing of the uploaded photographs with researchers, healthcare professionals and journalists for publication purposes.

### Statistical analyses

Statistical analyses were carried out using R, version 3.6.1 (R Foundation, Vienna, Austria). To identify confounders, comparisons between categorical and continuous values were carried out using Pearson’s χ^2^‐test and Wilcoxon’s test or linear regression, respectively. Associations between the presence or absence of self‐reported skin‐related symptoms and, in tested users, SARS‐CoV‐2 test results and, in symptomatic untested users, the presence or absence of the three classic COVID‐19 symptoms included in the UK NHS guidelines, were carried out through multivariate logistic regression. The following variables, identified in the previous analysis, were included as covariates: sex, age, BMI, ethnicity, smoking status (never, former, current), common diseases (diabetes and lung disease) and whether corticosteroids, immunosuppressants or blood pressure medications were administered. Associations passing a Bonferroni‐derived threshold of 0·05/2 (body rash and acral rash) = 0·025 were considered as significant. Sensitivity analyses were carried out by stratifying for sex, ethnicity and being a healthcare worker.

### Data and code sharing

Data collected in the app are being shared with other health researchers through the NHS‐funded Health Data Research UK (HDRUK)/SAIL consortium, housed in the UK Secure e‐Research Platform in Swansea. Anonymized data collected by the symptom tracker app can be shared with researchers who provide a methodologically sound proposal via HDRUK, provided the request is made according to their protocols and is in the public interest.^12^ Data updates can be found at https://covid.joinzoe.com. The app code is publicly available from https://github.com/zoe/covid‐tracker‐react‐native. The data screening script is publicly available from https://github.com/KCL‐BMEIS/zoe‐data‐prep.

## Results

### Cutaneous rashes are predictive of SARS‐CoV‐2 infection

Here, we investigated whether cutaneous manifestations are specific to SARS‐CoV‐2 infection in 336 847 UK users of the COVID Symptom Study app^11^ who registered between 7 May and 22 June 2020 (Figures S1 and S2). The majority of the users included in this study were white European (94·0%). Results for SARS‐CoV‐2 swab tests were provided by 27 157 users (8·1%), 2021 of whom (7·4%) were positive. Among untested users, 54 652 were symptomatic (i.e. they reported at least one of the 16 collected symptoms), including 17 371 users presenting with at least one of the three symptoms of COVID‐19 (i.e. fever, persistent cough and/or anosmia), whose presence, according to the UK NHS guidelines, would require isolation and testing. While these guidelines are not diagnostic, the presence of any of these three symptoms associates in our data with SARS‐CoV‐2‐positive swab with an odds ratio (OR) of 5·69, 95% confidence interval (CI) 5·13–6·31, *P* = 5.12×10^‐282^ 1 and Table S1 (see Supporting Information).

**Table 1:** Characteristics of the patients in the sample

	All users	Users tested for SARS‐CoV‐2				Symptomatic untested users			
		All	Positive	Negative	*P*‐value	All	With classic symptoms	Without classic symptoms	*P*‐value
Number	336 847	27 157	2021	25 136	–	54 652	17 371	37 281	–
Female	188 118 (55·8)	16 474 (60·7)	1376 (68·1)	15 098 (60·1)	1.5×10^‐12^	34 789 (63·7)	10 684 (61·5)	24 105 (64·7)	1.0×1010^‐12^
Age (years)^a^	43·9 (19·7)	43·9 (17·5)	43·9 (15·6)	43·9 (17·7)	0·09	41·4 (18·5)	38·2 (19·4)	42·9 (17·8)	1.5×1010^‐145^
BMI (kg m^−2^)^a^	26·2 (6·4)	27·0 (6·5)	28·2 (6·8)	26·9 (6·5)	3.8×1010^‐17^	26·6 (6·7)	26·7 (7·2)	26·5 (6·4)	2.0×1010^‐27^
Healthcare workers	31 915 (9·5)	7494 (27·6)	1190 (58·9)	6304 (25·1)	2.9×1010^‐234^	5344 (9·8)	1541 (8·9)	3803 (10·2)	1.2×1010^‐6^
Body rash	4812 (1·4)	1177 (4·3)	138 (6·8)	1039 (4·1)	1.1×1010^‐7^	2729 (5·0)	1128 (6·5)	1601 (4·3)	1.9×1010^‐20^
Acral rash	2188 (0·6)	520 (1·9)	62 (3·1)	458 (1·8)	5.9×1010^‐5^	1210 (2·2)	419 (2·4)	791 (2·1)	0·21

Categorical values are reported as number (percentage) and were compared using Pearson’s χ^2^‐test. ^a^Continuous values are reported as mean (SD) and were compared using Wilcoxon’s test. Association *P*‐values with body and acral rashes are from logistic regression, and with body mass index (BMI) from linear regression, adjusted for the relevant covariates. ‘Users tested’ refers to users self‐reporting a positive or negative swab test result. ‘Symptomatic untested users’ refers to users who reported at least one of the 16 collected symptoms, did not believe that they had already had COVID‐19 when first registering with the app, and had not yet been tested for SARS‐CoV‐2. ‘Classic symptoms’ refers to those included in the UK National Health Service guidelines (i.e. fever, persistent cough and/or anosmia).

Skin‐related symptoms were reported by 6403 users, including 1534 tested users, and by 3672 untested symptomatic users. Among the 2021 users who tested positive on swab test, 178 (8·8%) reported skin‐related changes. Of those, 138 (6·8%) reported body rashes and 62 (3·1%) acral rashes (Table 1). Only 22 (1·1%) reported both acral and body rashes. Infected users reporting acral rashes were slightly older (mean age 50·2 years) than those who did not (mean age 43·7 years; Wilcoxon’s test, *P* = 0·006). Additionally, the prevalence of body rashes was slightly higher among female participants (OR 1·60, 95% CI 1·08–2·44, *P* = 0·02). We did not observe any significant age difference for body rashes, or sex difference for acral rash prevalence (*P* > 0·05).

Similar skin symptoms were also seen in untested symptomatic users: 1429 (8·2%) users reporting any of the three main symptoms also reported a rash, compared with 6·0% for those who did not (OR 1·32, 95% CI 1·23–1·42, *P* = 4.7×10^‐15^).

Association analysis highlighted a higher prevalence of either body or acral rashes among users who tested positive for SARS‐CoV‐2 compared with those who tested negative (OR 1·67, 95% CI 1·42–1·97, *P* =  1.1×10^‐9^). The subtypes were similar: body rashes were associated with SARS‐CoV‐2‐positive swab with an OR of 1·66 (95% CI 1·37–1·99, *P* = 1.1×10^‐7^), whereas the OR for acral rashes was 1·74 (95% CI 1·33–2·28, *P* = 5.9×10^‐5^). In comparison, the OR for fever was 1·48 (95% CI 1·31–1·66, *P* = 4.3×10^‐11^). Sensitivity analyses confirmed the reported ORs (Tables S2 and S3, and Figures S4 and S5; see Supporting Information). Positive predictive values for all symptoms are reported in Table S4 (see Supporting Information).

The comparison between 17 371 symptomatic untested users who reported at least one of the main symptoms and those who did not yielded an OR of 1·46 (95% CI 1·35–1·58, *P* =1.9×10^‐20^) for body rash, while the association with the rarer acral rash was not significant (*P* = 0·21). We could not assess whether ethnicity affected the prevalence of cutaneous symptoms as the number of non‐European users with skin symptoms was too low (Tables S1–S3 and Figures S4 and S5).

### Cutaneous rashes are often the first or only symptom

To better investigate the duration of skin rashes and their timing in relation to other COVID‐19 symptoms, we collected data from 11 544 individuals who responded to an independent online survey on COVID‐19‐related skin rashes (Figure S3). The median age was 53 years (interquartile range 41–63) and 77% were female. In total, 694 reported a positive SARS‐CoV‐2 swab or antibody test, and 3109 were untested but reported having had one of the three classic symptoms included in the UK NHS guidelines. The 694 surveyees who reported having tested positive to SARS‐CoV‐2 via a swab or antibody test, with skin clinical signs, also reported other COVID‐19‐related symptoms, with fatigue (11%), headaches (9%), loss of smell (9%), fever (7%), muscle pain (6%), shortness of breath (6%) and persistent cough (6%) being the most common. Interestingly, while most surveyees declared skin changes to appear at the same time as other COVID‐19 symptoms (47%) or afterwards (35%), in 17% of the cases skin symptoms appeared before any other symptoms, and in 21% of the cases the rash was the only symptom. Similar estimates were obtained when focusing on the 3109 symptomatic untested participants, where 47%, 39% and 15% of surveyees declared having had a cutaneous rash during, after or before any other symptoms, respectively.

### Papular rashes are the most common, while urticaria is short‐lived

**Figure 2:**
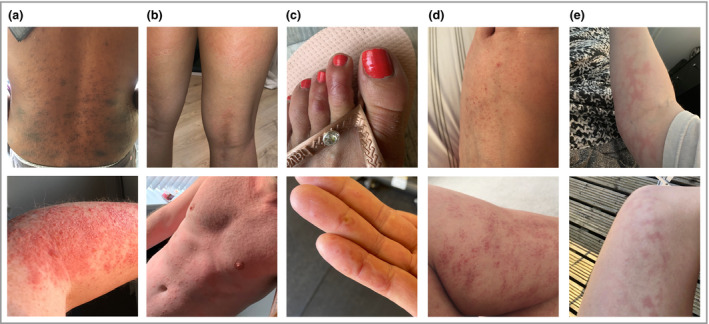
Example of COVID‐19‐related cutaneous manifestations. (a) Papular rash. Top: erythematopapular rash on the back. Bottom: erythematopapular eruption on the forearm; some blistering and necrosis of the top layers of the epidermis is also visible. (b) Urticarial rash. Top: large urticated plaques on the back of the thighs and popliteal fossae. Bottom: widespread urticaria on the torso. (c) Acral rash. Top: erythema on the dorsal aspect of the second and third toes with a blister on the second toe. Bottom: erythematous annular lesions with some shedding of the epidermis on the fingers and palms. (d) Vasculitic body. Top: petechiae on the dorsum of the foot. Bottom: multiple petechiae with blood cell extravasation on the calf. (e) Livedo reticularis. Top: livedo reticularis on the arm. Bottom: livedo reticularis on the thigh.

Photographs of rashes were shared by 2328 surveyees, and 260 photographs were randomly selected to be assessed by four experienced dermatologists, who divided them into five common categories: papular, urticarial, vasculitic body, livedo reticularis and acral lesions. We discarded 52 photographs (20%) rejected by at least one dermatologist because the image was blurred, or the area photographed was too small to evaluate, or the person could be de‐anonymized. Out of the 208 good‐quality photographs, 30 were judged as not attributable to SARS‐CoV‐2 infection (e.g. acne, shingles, molluscum contagiosum, pompholyx eczema, perioral dermatitis, impetigo or tinea; 14·4%), and 18 were discarded due to a disagreement in diagnosis between dermatologists (8·7%), resulting in 160 photographs used in the subsequent analyses. Notably, when asked whether a photograph showed a COVID‐19‐related skin rash, the four dermatologists agreed 82·2% of the time, and three out of four dermatologists agreed 95·7% of the time. The three most common presentations were papular rashes (including erythematopapular and erythematovesicular types, 41·2%), urticaria (30·0%) and acral lesions (23·1%). The overall levels of agreement for acral lesions and urticaria were 96·6% and 80·9%, respectively. Papular rashes showed the lowest agreement (76·4%), possibly because some the papular rashes may be more subtle and more difficult to photograph. Examples of COVID‐19 cutaneous manifestation are shown in Figure 2 and Figures S6–S8 (see Supporting Information). A large website, created in collaboration with the British Association of Dermatologists and including 400 manually curated high‐quality photographs, is available at https://covidskinsigns.com.

**Figure 3:**
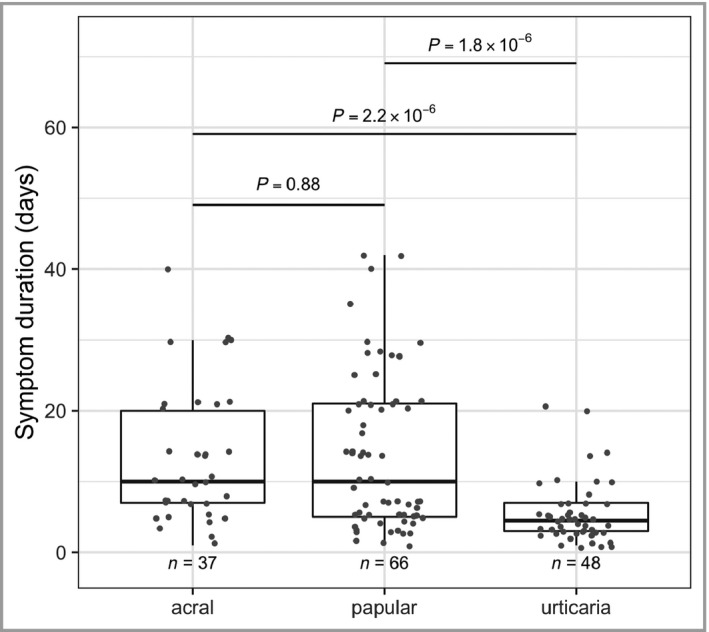
Duration of symptoms. Distribution of symptom duration is shown for the three most common cutaneous symptoms diagnosed from the selected photographs. *P*‐values were generated via Wilcoxon’s test.

The average duration of cutaneous rash was 13 days for acral lesions, 14 days for papular lesions and 5 days for urticaria (significantly shorter duration; Wilcoxon’s *P* < 2.3×10^‐6^; Figure 3).

## Discussion

COVID‐19 is now known to have varied clinical manifestations and to target multiple organs, including the skin.^1^, ^2^, ^4^ COVID‐19 rashes may present in many forms and at different stages of the disease.

In our app‐based study, 8·8% of patients who were positive for COVID‐19 via swab tests also reported skin rashes (OR 1·67, 95% CI 1·42–1·97). Body rashes were more frequent than acral lesions (6·8% vs. 3·1%), although their predictive value was similar (OR 1·66, 95% CI 1·37–1·99 vs. 1·74, 95% CI 1·33–2·28, respectively). Interestingly, the OR for both types of rash was greater than for fever (OR 1·48), a widely used criterion to suggest testing. Reports of cases with both body rashes and acral lesions were rare (1·1%), suggesting different pathogenesis, with the former caused by immunological reactions to the virus, and the latter more likely explained by delayed small thrombotic occlusions or damage to vessel walls.^4^

While we could not exclude that acral lesions were due to lifestyle changes,^13^ other studies observed acral lesions along with positive nasopharyngeal swab and/or serology for SARS‐CoV‐2.^14^, ^15^, ^16^, ^17^ Notably, a small study involving seven paediatric patients presenting with acral rashes found coronavirus‐like particles in the endothelial cells on electron microscopy, along with further histological evidence of vascular damage, supporting a causal relation of the lesions with SARS‐CoV‐2 infection.^18^

The use of the app has been valuable to document the presence of different types of COVID‐19 symptoms in the community.^19^, ^20^, ^21^ However, data on cutaneous symptoms were only recently collected, and this hindered our ability to identify at which stage of the disease they appear and how long they last. An independent survey was therefore carried out to capture more details on the types of rashes, including photographs, their duration and timing, results from SARS‐CoV‐2 swab or antibody test, and co‐occurring symptoms. The prevalence of the different types of rashes was assessed by four dermatologists using uploaded photographs. This showed that papular rashes were the most frequent and that urticaria was short‐lived. The survey also showed that in 17% of the positive surveyees, skin rash was the first symptom to appear, as already observed in two case reports.^8^, ^22^ Importantly, in 21% of them it was the only symptom, and a SARS‐CoV‐2 diagnosis would have been missed if using either the UK NHS or the US Centers for Disease Control and Prevention criteria alone.

A major limitation of this study is the self‐reported nature of the data. However, we believe that the presence of a rash, especially if symptomatic, is less subjective and more specific than other symptoms such as fatigue or headaches. Of the photographs uploaded by the surveyees and blindly assessed by four dermatologists, only 14% were classified definitively as dermatological conditions not related to COVID‐19, suggesting that the large majority of surveyees (86%) were able to self‐identify cutaneous manifestation likely to be related to COVID‐19 infection. This may mean that the total number of self‐reported COVID‐19‐related skin rashes was slightly overestimated. On the other hand, many of the app users may have failed to realize the relevance of cutaneous symptoms and not reported them if not accompanied by other more known COVID‐19 symptoms. Given the very large number of users of both the app and the survey, we are confident that potential errors deriving from the analysis of data reported by 336 847 users are likely reflected by larger standard errors of the estimates, rather than the point estimate of their effects, as already postulated in other large and successful biobank resources (e.g. the UK Biobank), which are also based on self‐reported health‐related data.

A second limitation is that our study sample is not fully representative of the general population, as it represents a self‐selected group of individuals. Our study sample is also composed predominantly of white individuals, and with larger proportions of female and younger individuals compared with those observed in hospital settings. Moreover, the data were usually collected only during the milder phases of the disease and in individuals who did not require hospitalization as, when health deteriorates, logging often stops.

Thirdly, we did not consider rare dermatological presentations. The main aim of this study was not to provide an exhaustive description of SARS‐CoV‐2 cutaneous manifestations but to raise awareness of the high prevalence of common COVID‐19 rashes, which can sometimes appear earlier than other COVID‐19 symptoms or be the only symptom. To this purpose, we have further developed a large library of curated photographs (https://covidskinsigns.com) to help general practitioners and healthcare professionals not specialized in dermatology to recognize the most common categories of COVID‐19 rash.

Fourthly, some of these cutaneous manifestations could have been caused by adverse reactions to drugs used to treat SARS‐CoV‐2 and/or for other purposes.^23^ Due to the size of our study and to the nature of the app used to collect our data, we could not collect a full history for drug usage, either at the time of data collection or retrospectively. However, we expect the numbers of community‐based users on drugs that could have also caused a rash to be very low compared with the number of rashes observed in hospitalized patients, and therefore drugs are unlikely to have been a major alternate cause of rashes. Similarly, we could not collect information on pre‐existing skin diseases, and could not investigate whether they influenced the rate of the reported rashes.

In summary, this study strongly supports the inclusion of skin rashes in the list of suspicious COVID‐19 symptoms. Although they are less prevalent than fever, they are more specific and last longer, and can be easily spotted by patients. Importantly from the survey reported in this study, it was the only presentation in one‐fifth of the patients diagnosed with COVID‐19.

Increased awareness from the public and healthcare professionals regarding COVID‐19 skin changes will allow more efficient detection of infection and contact tracing.

## Author Contribution

**Alessia Visconti:** Conceptualization (lead); Data curation (lead); Formal analysis (lead); Investigation (lead); Methodology (lead); Software (lead); Visualization (lead); Writing‐original draft (lead); Writing‐review & editing (lead). **V Bataille:** Conceptualization (lead); Data curation (lead); Investigation (lead); Methodology (lead); Writing‐original draft (lead); Writing‐review & editing (lead). **Niccolò Rossi:** Data curation (lead); Formal analysis (lead); Investigation (equal); Methodology (equal); Visualization (equal); Writing‐original draft (equal); Writing‐review & editing (equal). **Justine Kluk:** Data curation (equal); Writing‐review & editing (supporting). **Ruth Murphy:** Data curation (equal); Writing‐review & editing (supporting). **Susana Puig:** Data curation (equal); Writing‐review & editing (supporting). **Rabi Nambi:** Data curation (equal); Writing‐review & editing (supporting). **Ruth CE Bowyer:** Data curation (supporting); Writing‐review & editing (supporting). **Benjamin Murray:** Data curation (supporting); Software (lead); Writing‐review & editing (supporting). **Abigail Bournot:** Data curation (lead); Writing‐review & editing (supporting). **Jonathan Wolf:** Conceptualization (equal); Data curation (equal); Project administration (equal); Resources (equal); Writing‐review & editing (supporting). **Sebastien Ourselin:** Software (equal); Writing‐review & editing (supporting). **Claire J Steves:** Funding acquisition (equal); Writing‐review & editing (supporting). **tim. Spector:** Conceptualization (lead); Funding acquisition (lead); Investigation (lead); Supervision (lead); Writing‐original draft (lead); Writing‐review & editing (lead). **Mario Falchi:** Conceptualization (lead); Data curation (lead); Formal analysis (lead); Investigation (lead); Methodology (lead); Supervision (lead); Writing‐original draft (lead); Writing‐review & editing (lead).

## Supporting information


**Figure S1.** The protocol for selecting the study sample.
**Figure S2.** The protocol used to partition the selected COVID Symptom Study app users.
**Figure S3.** The protocol used for selecting the study sample from the survey data.
**Figure S4.** Sensitivity analysis for multivariate logistic regression in users tested for SARS‐CoV‐2 infection.
**Figure S5.** Sensitivity analysis for multivariate logistic regression in untested symptomatic users.
**Figure S6.** Examples of papular rash.
**Figure S7.** Examples of urticarial rash.
**Figure S8.** Examples of acral rash.
**Table S1.** Sample characteristics.
**Table S2.** Sensitivity analysis for multivariate logistic regression in users tested for SARS‐CoV‐2 infection.
**Table S3.** Sensitivity analysis for multivariate logistic regression in untested symptomatic users.
**Table S4.** Positive predictive values for all symptoms collected via the COVID Symptom Study app in users tested for SARS‐CoV‐2 infection.

**Powerpoint S1** Journal Club Slide Set.

**Video S1** Author video
