# Supplementary material for: Diagnostic value of cutaneous manifestation of SARS‐CoV‐2 infection*
Source: Br J Dermatol. 2021 May 1;184(5):880–7. doi: 10.1111/bjd.19807 (PMC8014275; doi:10.1111/bjd.19807)
Supplement: bjd19807-sup-0001-Supinfo — Figure S1. The protocol for selecting the study sample. Figure S2. The protocol used to partition the selected COVID Symptom Study app users. Figure S3. The protocol used for selecting the study sample from the survey data. Figure S4. Sensitivity analysis for multivariate logistic regression in users tested for SARS‐CoV‐2 infection. Figure S5. Sensitivity analysis for multivariate logistic regression in untested symptomatic users. Figure S6. Examples of papular rash. Figure S7. Examples of urticarial rash. Figure S8. Examples of acral rash. Table S1. Sample characteristics. Table S2. Sensitivity analysis for multivariate logistic regression in users tested for SARS‐CoV‐2 infection. Table S3. Sensitivity analysis for multivariate logistic regression in untested symptomatic users. Table S4. Positive predictive values for all symptoms collected via the COVID Symptom Study app in users tested for SARS‐CoV‐2 infection. [file bjd19807-sup-0001-supinfo.pdf]

Supplementary Material for:

Diagnostic value of cutaneous manifestation of SARS-CoV-2 infection

Alessia Visconti[1,*], Veronique Bataille[1,2,*], Niccolò Rossi[1], Justine Kluk[3], Ruth Murphy[4], Susana Puig[5,6], Rabi Nambi[7], Ruth C. E. Bowyer[1], Benjamin Murray[8], Abigail Bournot[3], Jonathan Wolf[3], Sebastien Ourselin[8], Claire J. Steves[1], Tim D Spector[1,†], and Mario Falchi[1,†]

[1]Department of Twin Research & Genetic Epidemiology, King's College London, London, UK
[2]Dermatology Department, West Herts NHS Trust, Herts, UK
[3]Zoe Global Limited, London, UK
[4]Dermatology Department, Sheffield Teaching Hospitals NHS Foundation Trust, Sheffield, UK
[5]Dermatology Department, Hospital Clinic of Barcelona, University of Barcelona, Barcelona, Spain
[6]Institut d'Investigacions Biomèdiques August Pi I Sunyer, Barcelona, Spain
[7]University Hospitals of Derby and Burton NHS Foundation Trust, Derby, UK
[8]School of Biomedical Engineering & Imaging Sciences, King's College London, London, UK
*These authors contributed equally
[†]These authors jointly supervised this work

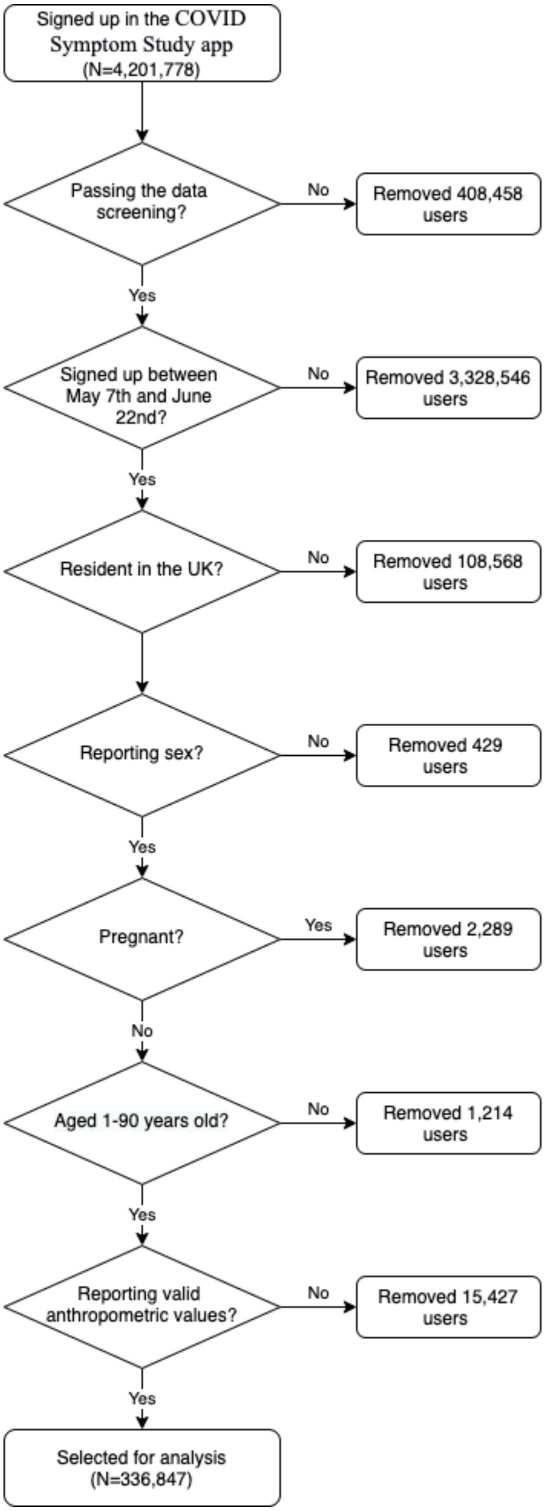

Supplementary Figure S1. The flowchart depicts the protocol used for selecting the study sample from the COVID Symptom Study app data. The data screening was carried out using a modified version of the *zoe-data-prep* python script (https://github.com/KCL-BMEIS/zoe-data-prep, version 0.1.9) which was used to retain users satisfying the following criteria: logged at least once, age range between 0 and 120 years old, height between 0.2 and 2.2 m, weight between 3 and 200 kg, and body mass index (BMI) between 0 and 55 kg/m^2. Valid anthropometric values were: for users 16 years old or older: height, weight, or BMI within the range of 1.1 to 2.2 m, 40 to 200 kg, and 15 to 55 kg/m^2, respectively; for users younger than 16 years old: height, weight or BMI within two standard deviations from the sample's mean for each age.

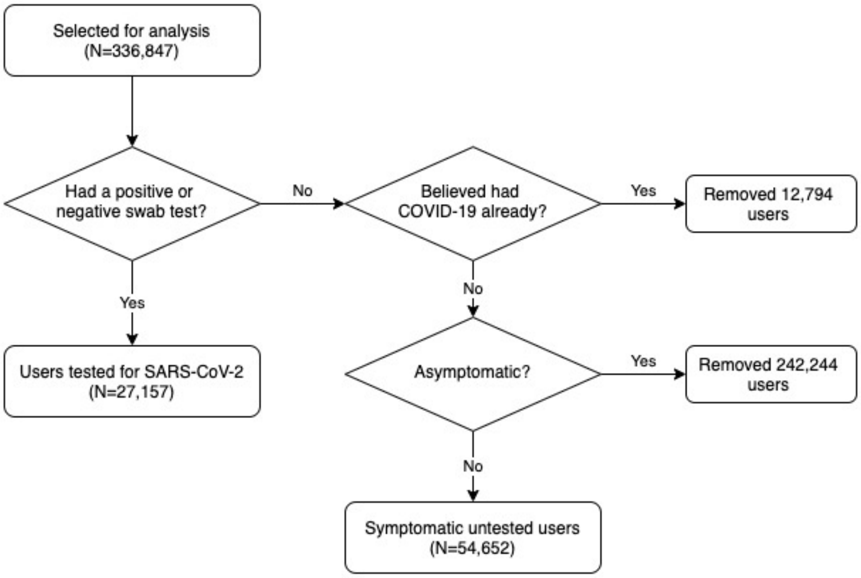

Supplementary Figure S2. The flowchart depicts the protocol used to partition the selected COVID Symptom Study app users (see Supplementary Figure 1 for details on sample selection) in users tested for SARS-CoV-2 and in untested asymptomatic users.

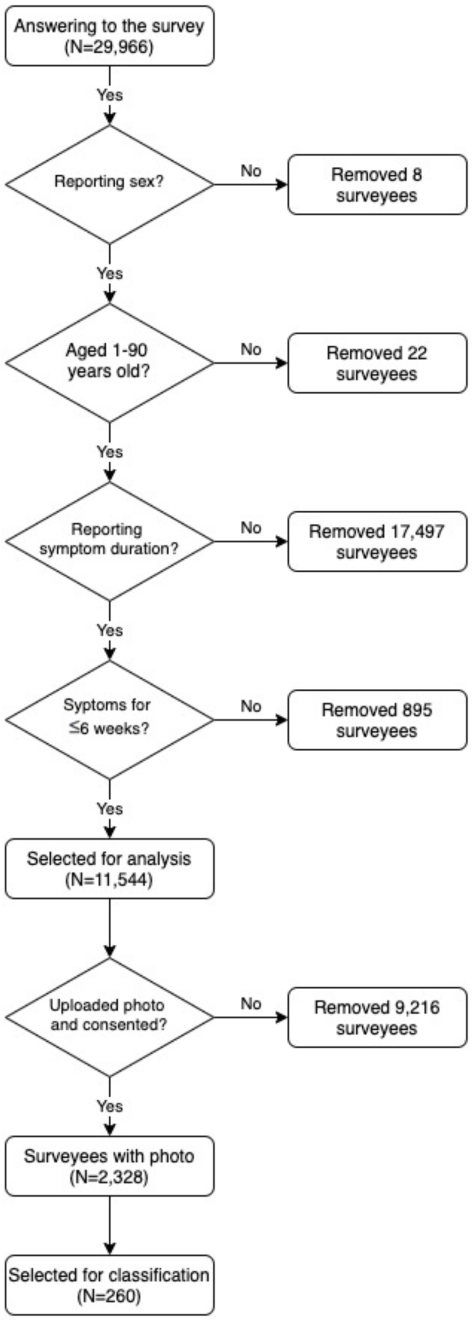

Supplementary Figure S3. The flowchart depicts the protocol used for selecting the study sample from the survey data.

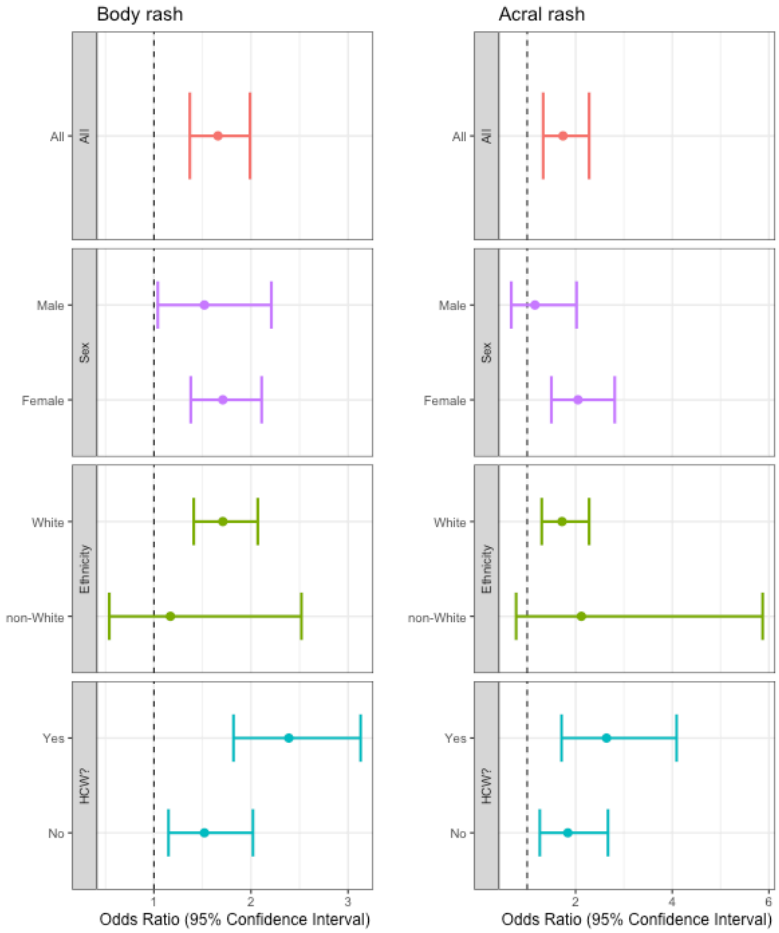

Supplementary Figure S4. Sensitivity analysis for multivariate logistic regression in users tested for SARS-CoV-2 infection. Forest plots showing the OR of self-reporting a skin-related symptom in users tested for SARS-CoV-2 infection, stratified by sex, ethnicity, and being a healthcare worker (HCW). The whiskers represent the 95% confidence interval.

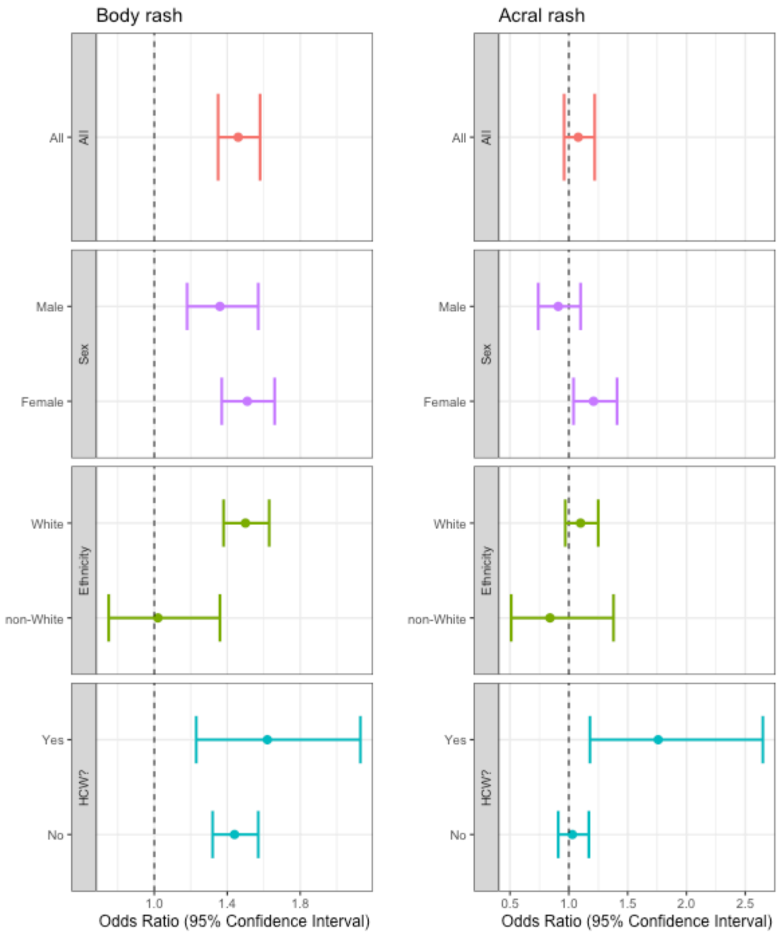

Supplementary Figure S5. Sensitivity analysis for multivariate logistic regression in untested symptomatic users. Forest plots showing the OR of self-reporting a skin-related symptom in users while experiencing at least one of the three classic symptoms included in the UK NHS guidelines (*i.e.,* fever, persistent cough, and/or anosmia) stratified by sex, ethnicity, and being a healthcare worker (HCW). The whiskers represent the 95% confidence interval.

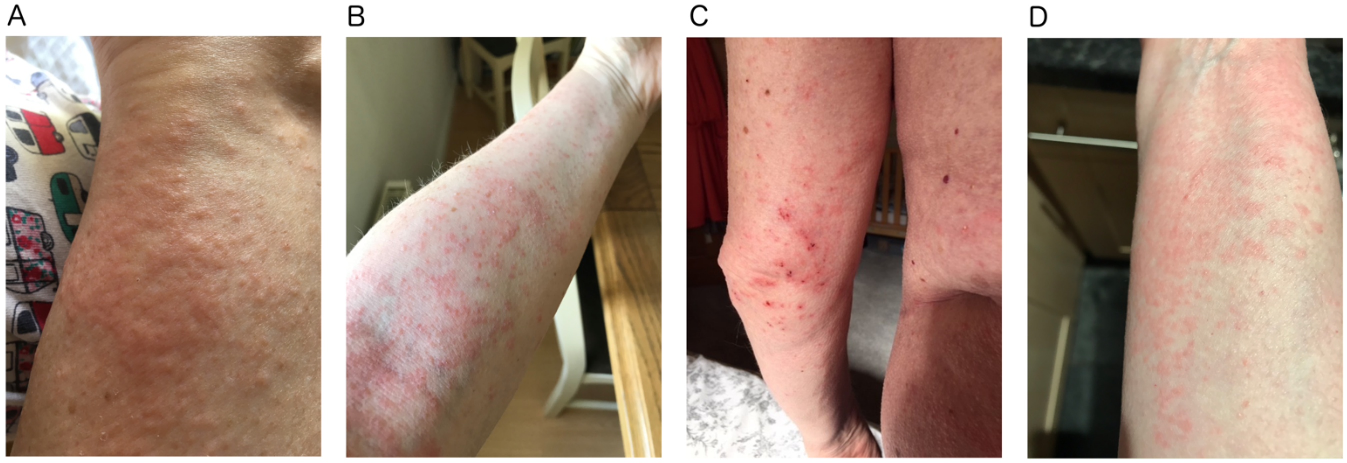

Supplementary Figure S6. Examples of papular rash. (A) Erythematopapular rash on forearm. (B) Erythematopapular rash on arm. (C) Erythematopapular rash with some dried vesicles on the elbows. (D) Erythematopapular rash on the forearm.

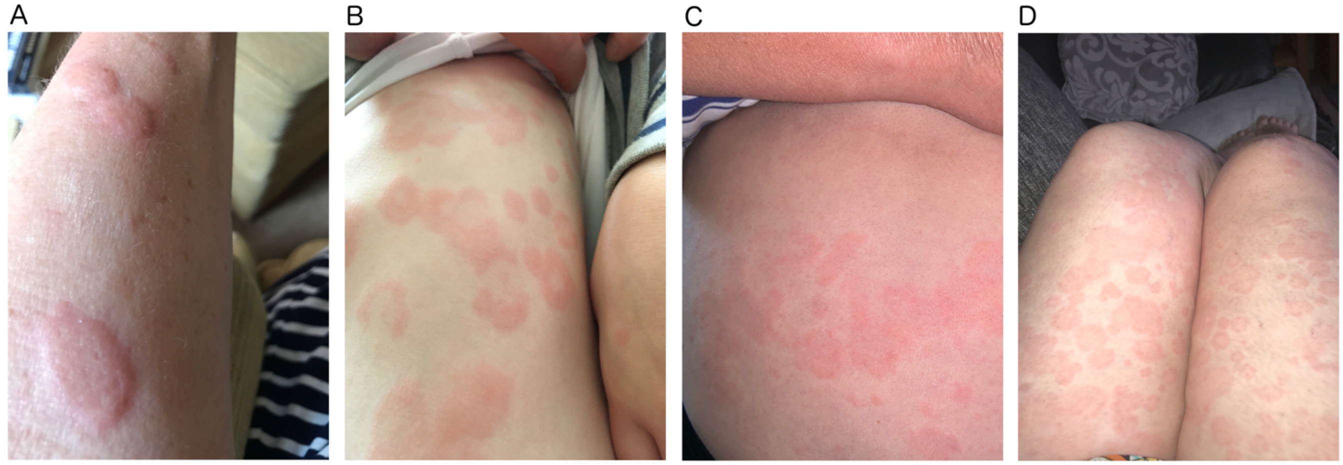

Supplementary Figure S7. Examples of urticarial rash. (A) Tumid urticated plaques on arm. (B) Urticated plaques on the abdomen with annular lesions. (C) Urticarial rash on abdomen. (D) Widespread urticaria on the thighs.

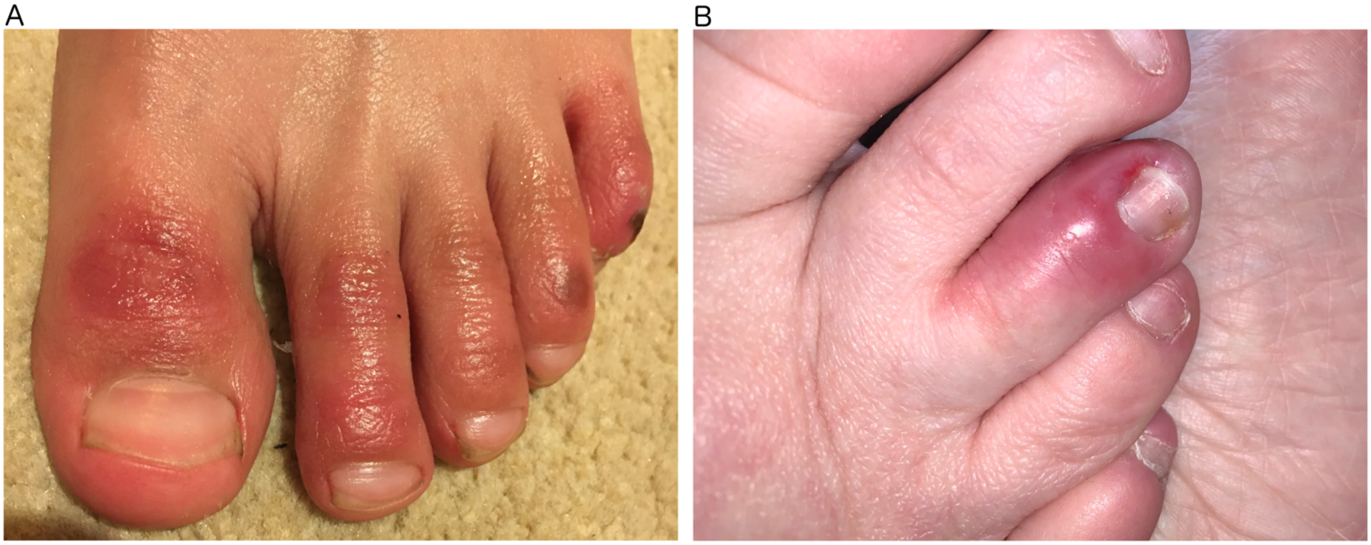

Supplementary Figure S8. Examples of acral rash. (A) Erythema on the dordal aspects of all toes with some epidermal necrosis on the 4th and 5th toe. (B) Swelling of the toe with erythema.

Supplementary Tables

Supplementary Table S1. Sample characteristics. Values are reported as number and percentage. Ethnicity, smoking, disease status and medications were compared using Pearson's χ^2 test, as implemented in the *prop.test* function (stats R package, v 3.6.0). "Users tested for SARS-CoV-2" refers to users self-reporting a positive or negative swab test result. "Symptomatic untested users" refers to users who reported at least one of the 16 collected symptoms, did not believe that they had already had COVID-19 when registering with the app, and had not been tested for SARS-CoV-2. "Classic symptoms" refer to those included in the NHS guidelines (*i.e.,* fever, persistent cough, and/or anosmia), whose presence would require isolation and testing for SARS-CoV-2 infection.

		All users	Users tested for SARS-CoV-2				Symptomatic untested users			
			All	Positive	Negative	P	All	With classical symptoms	Without classic symptoms	P
N		336,847	27,157	2,021	25,136		54,652	17,371	37,281	
Ethnicity						4.72×10^{-6}				$<2 \times 10^{-16}$
	Asian	6,503 (1.9%)	646 (2.4%)	70 (3.5%)	576 (2.3%)		1,029 (1.9%)	422 (2.4%)	607 (1.6%)	
	Black	2,105 (0.6%)	232 (0.9%)	34 (1.7%)	198 (0.8%)		370 (0.7%)	143 (0.8%)	227 (0.6%)	
	Chinese	1,029 (0.3%)	87 (0.3%)	6 (0.3%)	81 (0.3%)		134 (0.2%)	43 (0.2%)	91 (0.2%)	
	Middle East	1,204 (0.4%)	114 (0.4%)	13 (0.6%)	101 (0.4%)		187 (0.3%)	84 (0.5%)	103 (0.3%)	
	Mixed	6,974 (2.1%)	526 (1.9%)	35 (1.7%)	491 (2.0%)		1,295 (2.4%)	508 (2.9%)	787 (2.1%)	
	White	316,567 (94.0%)	25,341 (93.3%)	1,840 (91.0%)	23,5012 (93.5%)		51,188 (93.7%)	16,021 (92.2%)	35,167 (94.3%)	
	N/A	2,465 (0.7%)	211 (0.8%)	23 (1.1%)	188 (0.7%)		449 (0.8%)	150 (0.9%)	299 (0.8%)	
Smoking status						0.04				$<2 \times 10^{-16}$
	Never	241,093 (71.6%)	18,499 (68.1%)	1,396 (69.1%)	17,103 (68.0%)		37,932 (69.4%)	11,752 (67.7%)	26,180 (70.2%)	
	Ex	62,563 (18.6%)	5,655 (20.8%)	436 (21.6%)	5,219 (20.8%)		10,726 (19.6%)	3,193 (18.4%)	7,533 (20.2%)	
	Current	33,176 (9.8%)	3,002 (11.1%)	189 (9.4%)	2,813 (11.2%)		5,994 (11.0%)	2,426 (14.0%)	3,568 (9.6%)	

Has diabetes (%)	12,884 (3.8%)	1,1191 (4.4%)	113 (5.6%)	1,078 (4.3%)	$7.04x10^{-3}$	2,109 (3.9%)	704 (4.1%)	1,405 (3.8%)	0.11
Has heart disease (%)	10,052 (3.0%)	936 (3.4%)	78 (3.9%)	858 (3.4%)	0.32	1,663 (3.0%)	529 (3.0%)	1,134 (3.0%)	1.00
Has lung disease (%)	31,489 (9.3%)	3,150 (11.6%)	264 (13.1%)	2,886 (11.5%)	$3.58x10^{-2}$	6,912 (12.6%)	2,603 (15.0%)	4,309 (11.6%)	$3.71x10^{-29}$
Has kidney disease (%)	2,598 (0.8%)	293 (1.1%)	29 (1.4%)	264 (1.1%)	0.13	527 (1.0%)	169 (1.0%)	358 (1.0%)	0.93
Has cancer (%)	4,456 (1.3%)	543 (2.0%)	34 (1.7%)	509 (2.0%)	0.33	660 (1.2%)	210 (1.2%)	450 (1.2%)	1.00
Corticosteroids (%)	24,583 (7.3%)	2,491 (9.2%)	190 (9.4%)	2,301 (9.2%)	0.74	5,601 (10.2%)	2,028 (11.7%)	3,573 (9.6%)	$6.95x10^{-14}$
Immunosuppressants (%)	12,006 (3.6%)	1,138 (4.2%)	75 (3.7%)	1,063 (4.2%)	0.29	2,415 (4.4%)	910 (5.2%)	1,505 (4.0%)	$2.26x10^{-10}$
Blood pressure medications (%)	44,061 (13%)	3,593 (13.2%)	273 (13.5%)	3,320 (13.2%)	0.73	6,272 (11.5%)	1.852 (10.7%)	4,420 (11.9%)	$4.80x10^{-5}$

Supplementary Table S2. Sensitivity analysis for multivariate logistic regression in users tested for SARS-CoV-2 infection. Associations between the presence/absence of self-reported skin-related symptoms and test results in users tested for SARS-CoV-2 infection, stratified by sex, ethnicity and being a healthcare worker, were carried out through multivariate logistic regression, and the following variables were included as covariates: sex, age, BMI, ethnicity (namely: Asian, Black, Chinese, Middle Eastern, White, or mixed), smoking status (namely: never, ex, current), common disease status (namely: diabetes and lung disease), and whether corticosteroids, immunosuppressants, or blood pressure medications were administered.

				**Body rash**			**Acral Rash**	
**Risk factor**	**Stratum**	**N**	**OR**	**95% CI**	**P**	**OR**	**95% CI**	**P**
	All sample	26,945	1.66	1.37-1.99	$1.06x10^{-7}$	1.74	1.33-2.28	$5.91x10^{-5}$
Sex	Male	10,598	1.52	1.04-2.21	0.03	1.16	0.67-2.02	0.590
	Female	16,347	1.71	1.38-2.11	$1.11x10^{-6}$	2.05	1.50-2.81	$7.69x10^{-6}$
Ethnicity	White	25,340	1.71	1.41-2.07	$4.51x10^{-8}$	1.72	1.30-2.28	$1.71x10^{-4}$
	Non-White	1,605	1.17	0.54-2.52	0.698	2.12	0.77-5.87	0.146
Healthcare	Yes	7,429	2.39	1.82-3.13	$2.87x10^{-10}$	2.64	1.71-4.09	$1.27x10^{-5}$
worker?	No	19,516	1.52	1.15-2.02	$3.44x10^{-3}$	1.84	1.26-2.67	$1.45x10^{-3}$

Supplementary Table S3. Sensitivity analysis for multivariate logistic regression in untested symptomatic users. Associations between the presence/absence of self-reported skin-related symptoms and presence/absence of at least one the three classic symptoms included in the NHS guidelines (*i.e.,* fever, persistent cough, and/or anosmia) in untested symptomatic users, stratified by sex, ethnicity and being a healthcare worker, were carried out through multivariate logistic regression, and the following variables were included as covariates: sex, age, BMI, ethnicity (namely: Asian, Black, Chinese, Middle Eastern, White, or mixed), smoking status (namely: never, ex, current), common disease status (namely: diabetes and lung disease), and whether corticosteroids, immunosuppressants, or blood pressure medications were administered.

				**Body rash**			**Acral Rash**	
**Risk factor**	**Stratum**	**N**	**OR**	**95% CI**	**P**	**OR**	**95% CI**	**P**
	All sample	54,203	1.46	1.35-1.58	$1.94x10^{-20}$	1.08	0.96 1.22	0.205
Sex	Male	19,697	1.36	1.18-1.57	$1.46x10^{-5}$	0.91	0.74 1.10	0.331
	Female	34,506	1.51	1.37-1.66	$2.34x10^{-16}$	1.21	1.04 1.41	0.016
Ethnicity	White	51,188	1.50	1.38-1.63	$6.36x10^{-22}$	1.10	0.97 1.25	0.135
	Non-White	3,015	1.02	0.75-1.36	0.921	0.84	0.51 1.38	0.486
Healthcare	Yes	5,298	1.62	1.23-2.13	$5.34x10^{-4}$	1.76	1.18 2.65	$6.12x10^{-3}$
worker?	No	48,905	1.44	1.32-1.57	$1.47x10^{-17}$	1.03	0.91 1.17	0.631

Supplementary Table S4. Positive predictive values (PPV) for all symptoms collected *via* the COVID Symptom Study app in users tested for SARS-CoV-2 infection.

**Symptom**	**PPV**
Anosmia	0.321
Shortness of breath	0.173
Persistent cough	0.158
Skipped meals	0.154
Chest pain	0.144
Hoarse voice	0.144
Unusual muscle pains	0.140
Acral rash	0.119
Fatigue	0.118
Delirium	0.117
Body rash	0.117
Headache	0.101
Fever	0.100
Diarrhoea	0.094
Abdominal pain	0.090
Sore throat	0.081
