# Supplementary material for: Diagnostic value of cutaneous manifestation of SARS‐CoV‐2 infection*
Source: Br J Dermatol. 2021 May 1;184(5):880–7. doi: 10.1111/bjd.19807 (PMC8014275; doi:10.1111/bjd.19807)
Supplement: bjd19807-sup-0002-JournalClub — Powerpoint S1 Journal Club Slide Set. [file bjd19807-sup-0002-journalclub.pptx]

## Slide 1
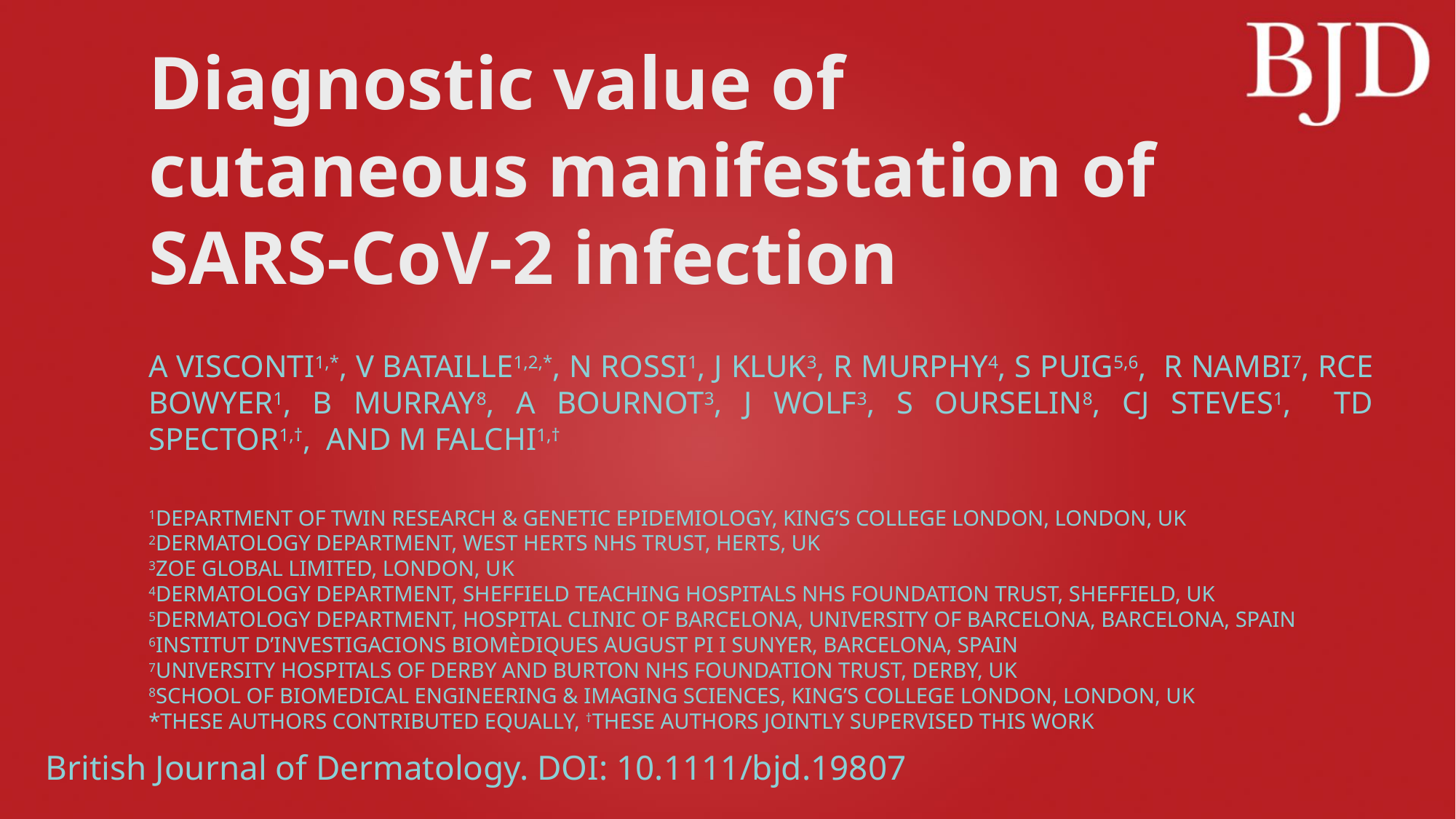

# Diagnostic value of cutaneous manifestation of SARS-CoV-2 infection
A Visconti1,*, V Bataille1,2,*, N Rossi1, J Kluk3, R Murphy4, S Puig5,6, R Nambi7, RCE Bowyer1, B Murray8, A Bournot3, J Wolf3, S Ourselin8, CJ Steves1, TD Spector1,†, and M Falchi1,†
1Department of Twin Research & Genetic Epidemiology, King’s College London, London, UK
2Dermatology Department, West Herts NHS Trust, Herts, UK
3Zoe Global Limited, London, UK
4Dermatology Department, Sheffield Teaching Hospitals NHS Foundation Trust, Sheffield, UK
5Dermatology Department, Hospital Clinic of Barcelona, University of Barcelona, Barcelona, Spain
6Institut d’Investigacions Biomèdiques August Pi I Sunyer, Barcelona, Spain
7University Hospitals of Derby and Burton NHS Foundation Trust, Derby, UK
8School of Biomedical Engineering & Imaging Sciences, King’s College London, London, UK
*These authors contributed equally, †These authors jointly supervised this work
British Journal of Dermatology. DOI: 10.1111/bjd.19807

## Slide 2
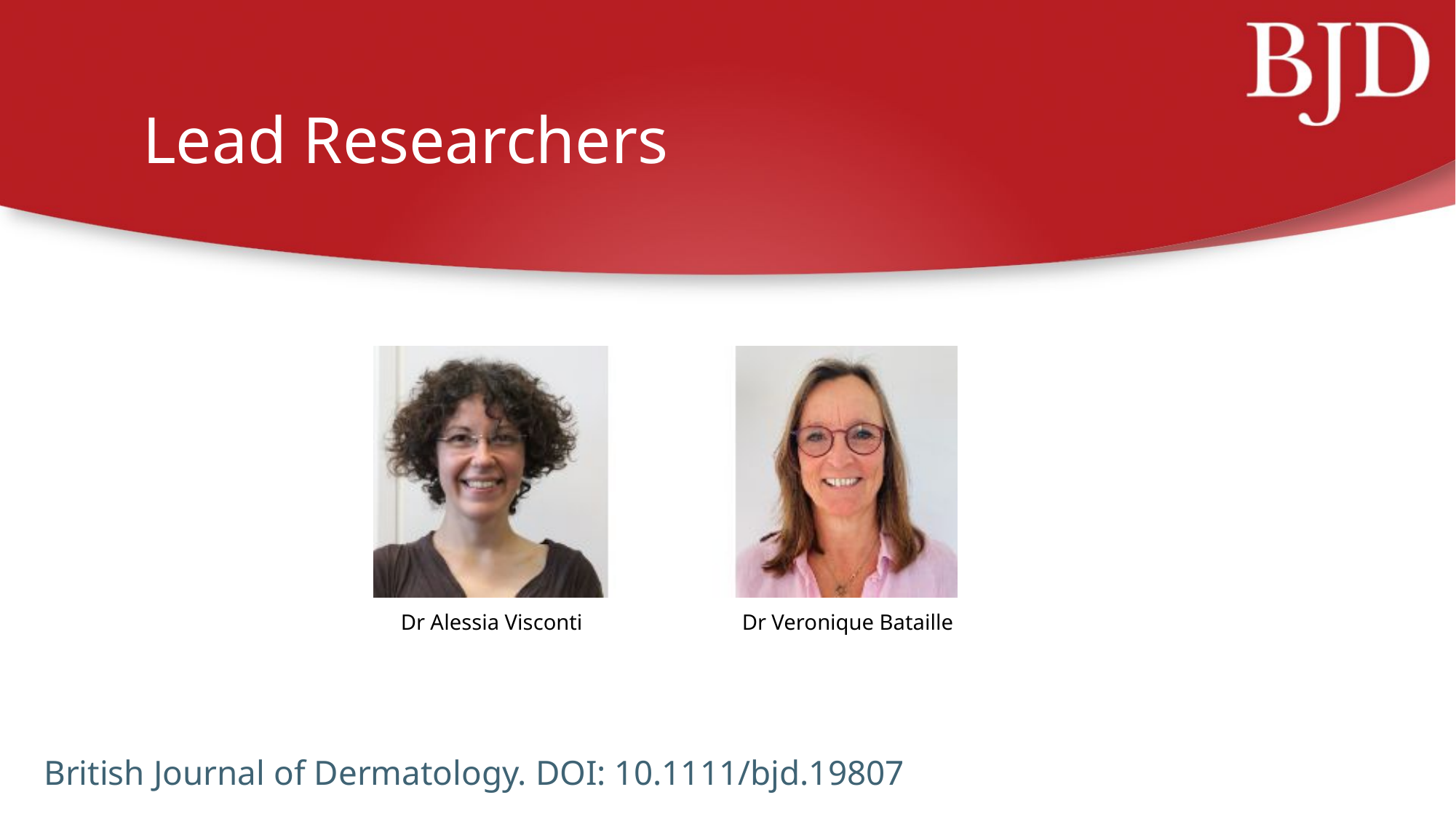

# Lead Researchers
Dr Alessia Visconti
Dr Veronique Bataille
British Journal of Dermatology. DOI: 10.1111/bjd.19807

## Slide 3
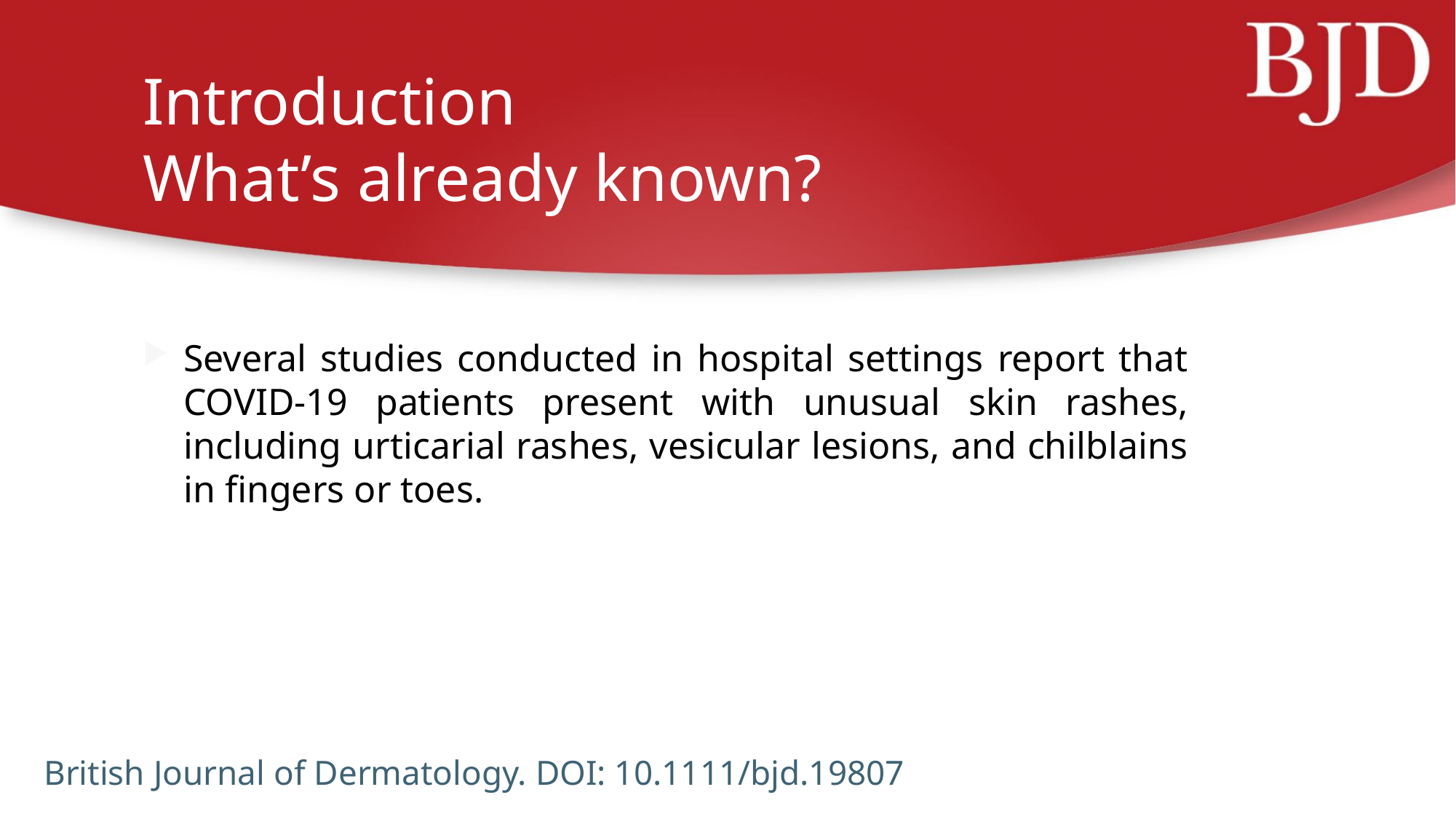

# Introduction What’s already known?
Several studies conducted in hospital settings report that COVID-19 patients present with unusual skin rashes, including urticarial rashes, vesicular lesions, and chilblains in fingers or toes.
British Journal of Dermatology. DOI: 10.1111/bjd.19807

## Slide 4
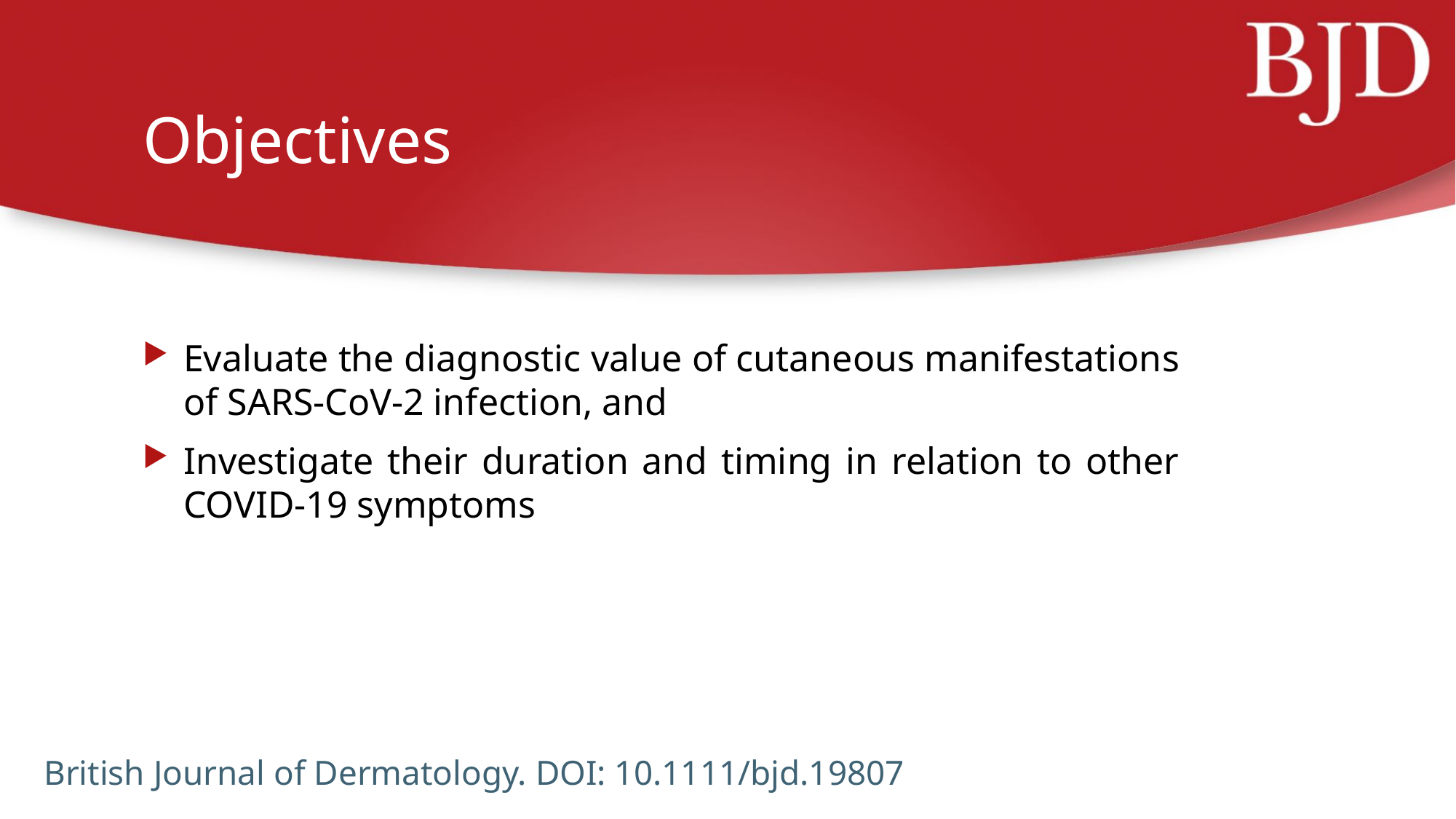

# Objectives
Evaluate the diagnostic value of cutaneous manifestations of SARS-CoV-2 infection, and
Investigate their duration and timing in relation to other COVID-19 symptoms
British Journal of Dermatology. DOI: 10.1111/bjd.19807

## Slide 5
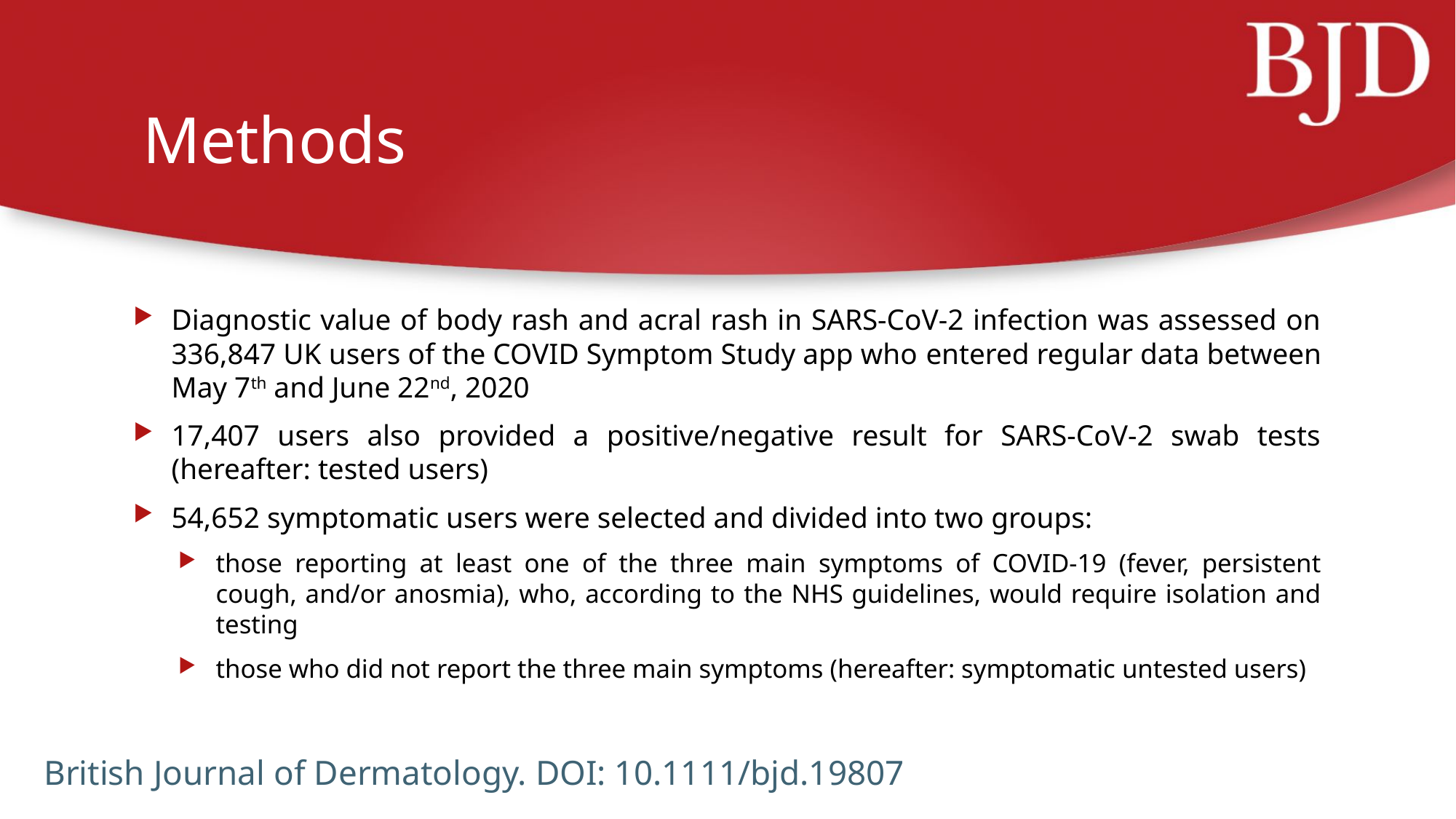

# Methods
Diagnostic value of body rash and acral rash in SARS-CoV-2 infection was assessed on 336,847 UK users of the COVID Symptom Study app who entered regular data between May 7th and June 22nd, 2020
17,407 users also provided a positive/negative result for SARS-CoV-2 swab tests (hereafter: tested users)
54,652 symptomatic users were selected and divided into two groups:
those reporting at least one of the three main symptoms of COVID-19 (fever, persistent cough, and/or anosmia), who, according to the NHS guidelines, would require isolation and testing
those who did not report the three main symptoms (hereafter: symptomatic untested users)
British Journal of Dermatology. DOI: 10.1111/bjd.19807

## Slide 6
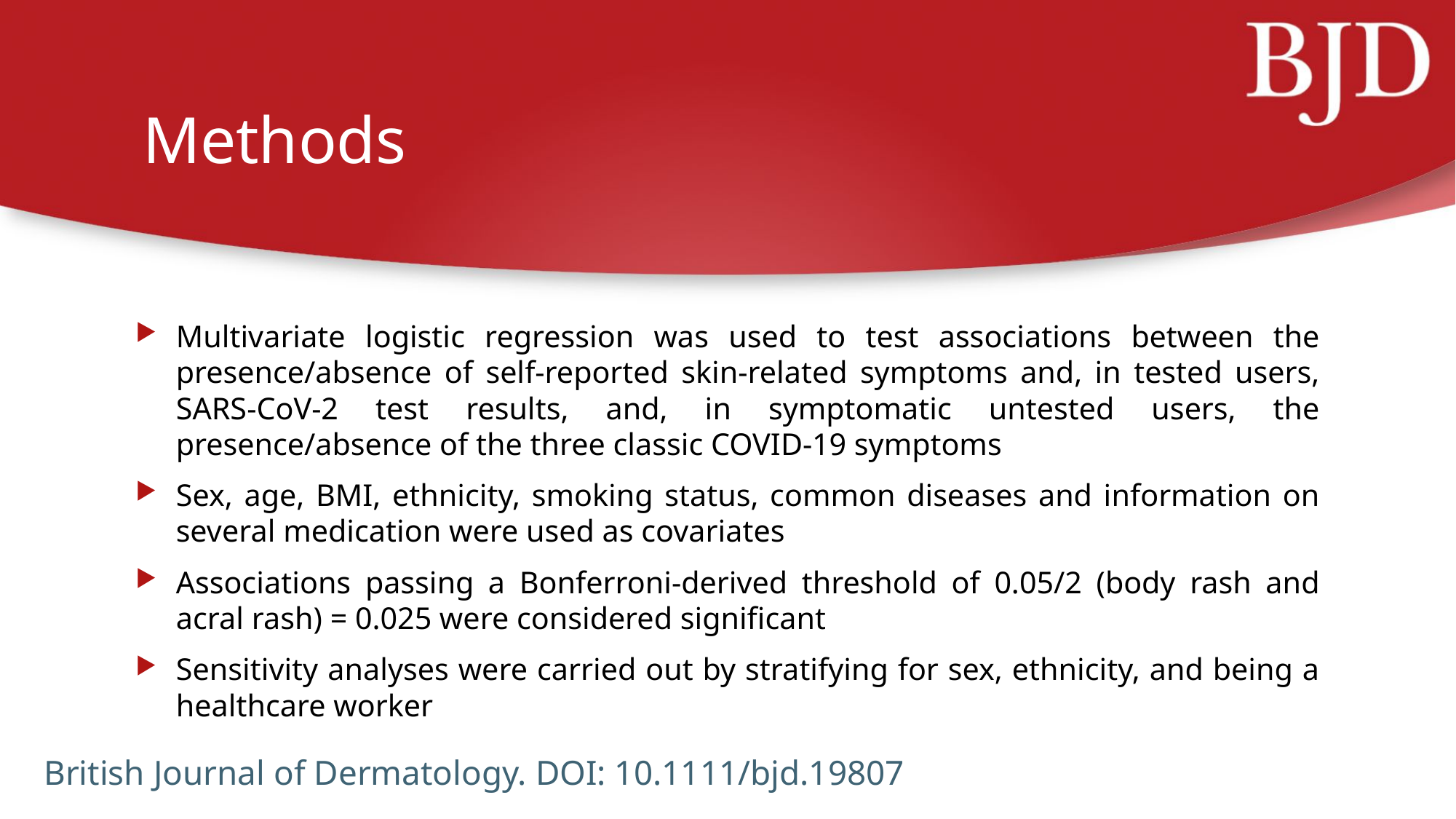

# Methods
Multivariate logistic regression was used to test associations between the presence/absence of self-reported skin-related symptoms and, in tested users, SARS-CoV-2 test results, and, in symptomatic untested users, the presence/absence of the three classic COVID-19 symptoms
Sex, age, BMI, ethnicity, smoking status, common diseases and information on several medication were used as covariates
Associations passing a Bonferroni-derived threshold of 0.05/2 (body rash and acral rash) = 0.025 were considered significant
Sensitivity analyses were carried out by stratifying for sex, ethnicity, and being a healthcare worker
British Journal of Dermatology. DOI: 10.1111/bjd.19807

## Slide 7
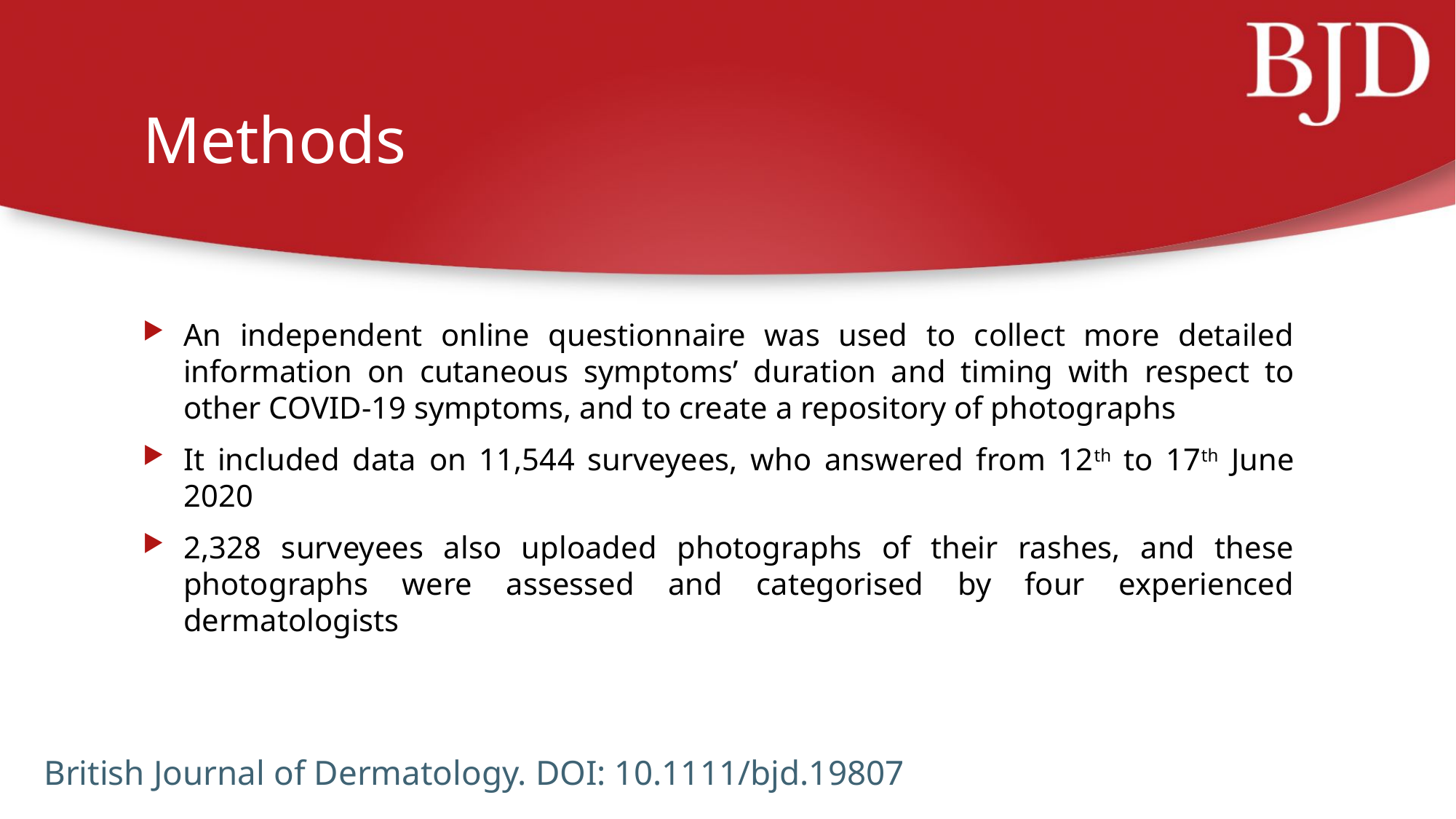

# Methods
An independent online questionnaire was used to collect more detailed information on cutaneous symptoms’ duration and timing with respect to other COVID-19 symptoms, and to create a repository of photographs
It included data on 11,544 surveyees, who answered from 12th to 17th June 2020
2,328 surveyees also uploaded photographs of their rashes, and these photographs were assessed and categorised by four experienced dermatologists
British Journal of Dermatology. DOI: 10.1111/bjd.19807

## Slide 8
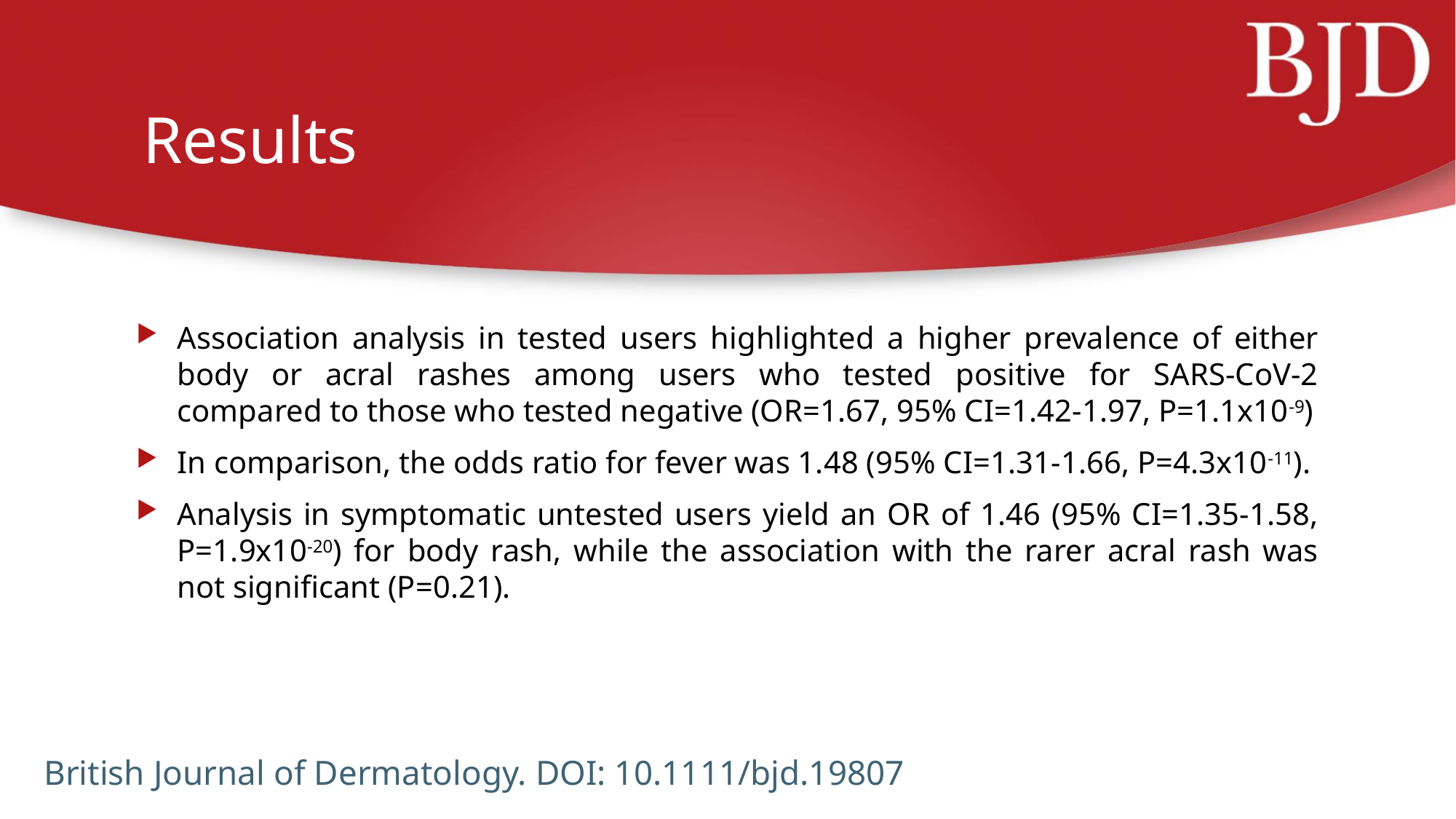

# Results
Association analysis in tested users highlighted a higher prevalence of either body or acral rashes among users who tested positive for SARS-CoV-2 compared to those who tested negative (OR=1.67, 95% CI=1.42-1.97, P=1.1x10^-9^)
In comparison, the odds ratio for fever was 1.48 (95% CI=1.31-1.66, P=4.3x10^-11^).
Analysis in symptomatic untested users yield an OR of 1.46 (95% CI=1.35-1.58, P=1.9x10^-20^) for body rash, while the association with the rarer acral rash was not significant (P=0.21).
British Journal of Dermatology. DOI: 10.1111/bjd.19807

## Slide 9
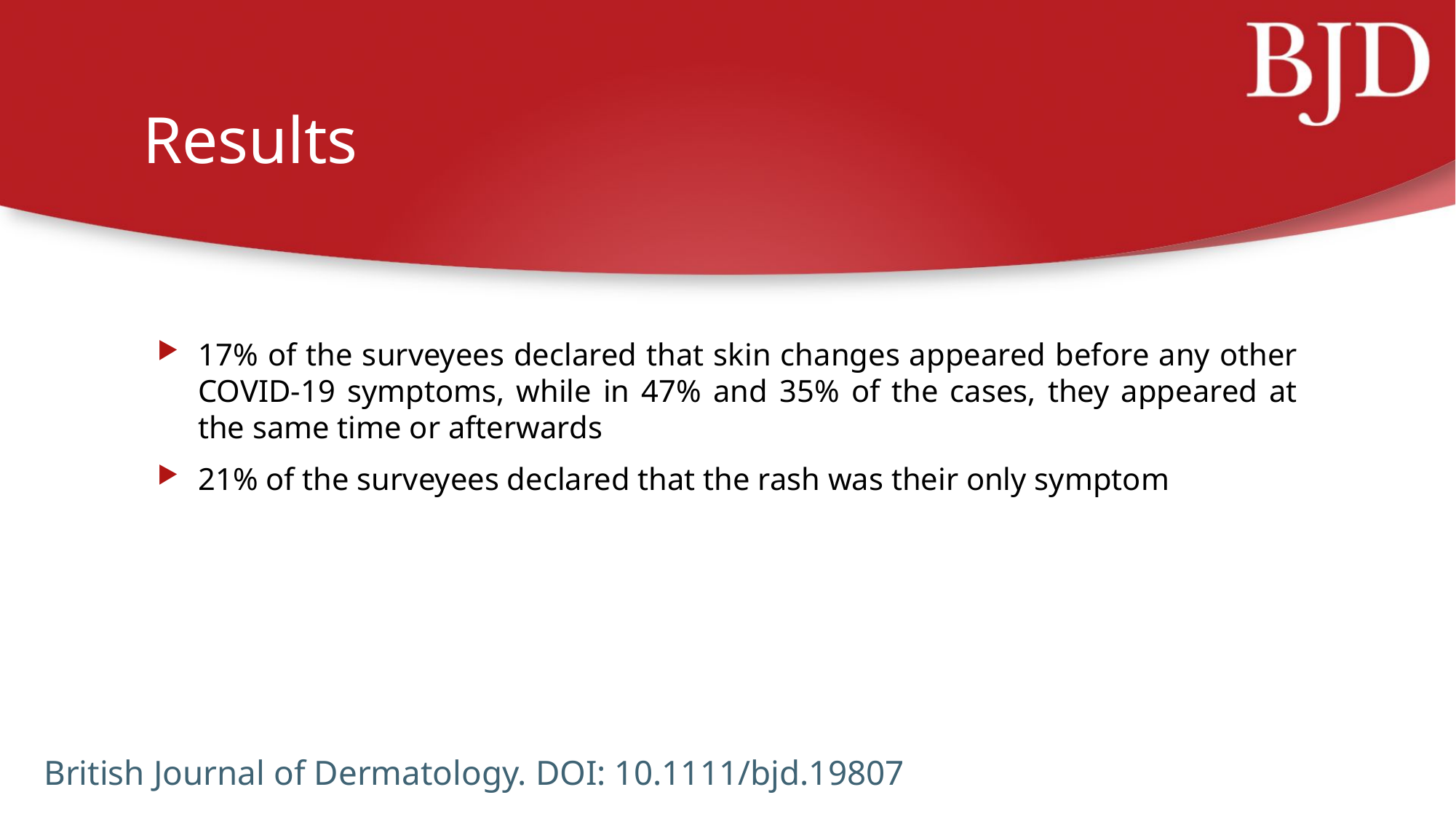

# Results
17% of the surveyees declared that skin changes appeared before any other COVID-19 symptoms, while in 47% and 35% of the cases, they appeared at the same time or afterwards
21% of the surveyees declared that the rash was their only symptom
British Journal of Dermatology. DOI: 10.1111/bjd.19807

## Slide 10
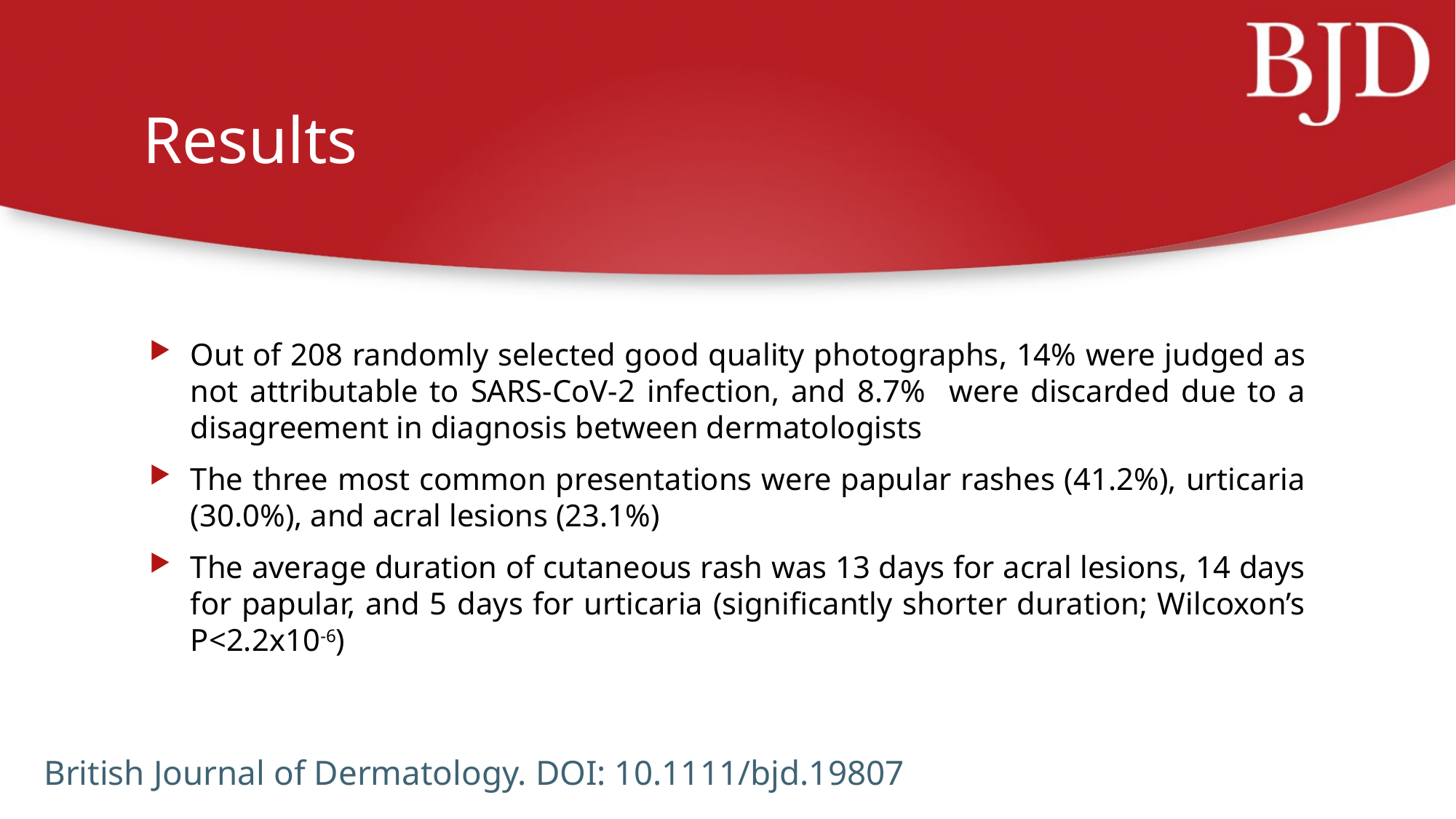

# Results
Out of 208 randomly selected good quality photographs, 14% were judged as not attributable to SARS-CoV-2 infection, and 8.7% were discarded due to a disagreement in diagnosis between dermatologists
The three most common presentations were papular rashes (41.2%), urticaria (30.0%), and acral lesions (23.1%)
The average duration of cutaneous rash was 13 days for acral lesions, 14 days for papular, and 5 days for urticaria (significantly shorter duration; Wilcoxon’s P<2.2x10^-6^)
British Journal of Dermatology. DOI: 10.1111/bjd.19807

## Slide 11
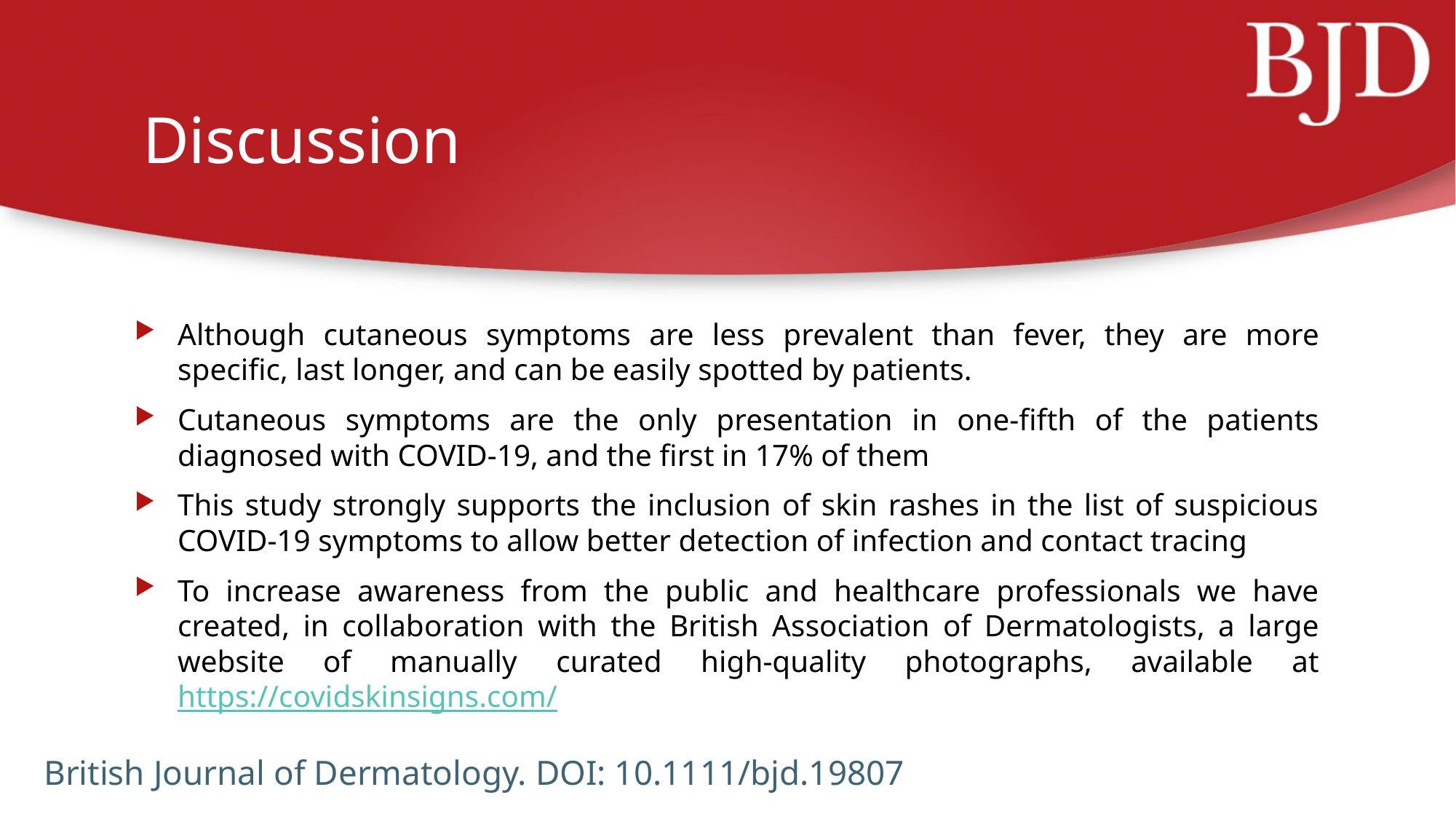

# Discussion
Although cutaneous symptoms are less prevalent than fever, they are more specific, last longer, and can be easily spotted by patients.
Cutaneous symptoms are the only presentation in one-fifth of the patients diagnosed with COVID-19, and the first in 17% of them
This study strongly supports the inclusion of skin rashes in the list of suspicious COVID-19 symptoms to allow better detection of infection and contact tracing
To increase awareness from the public and healthcare professionals we have created, in collaboration with the British Association of Dermatologists, a large website of manually curated high-quality photographs, available at https://covidskinsigns.com/
British Journal of Dermatology. DOI: 10.1111/bjd.19807

## Slide 12
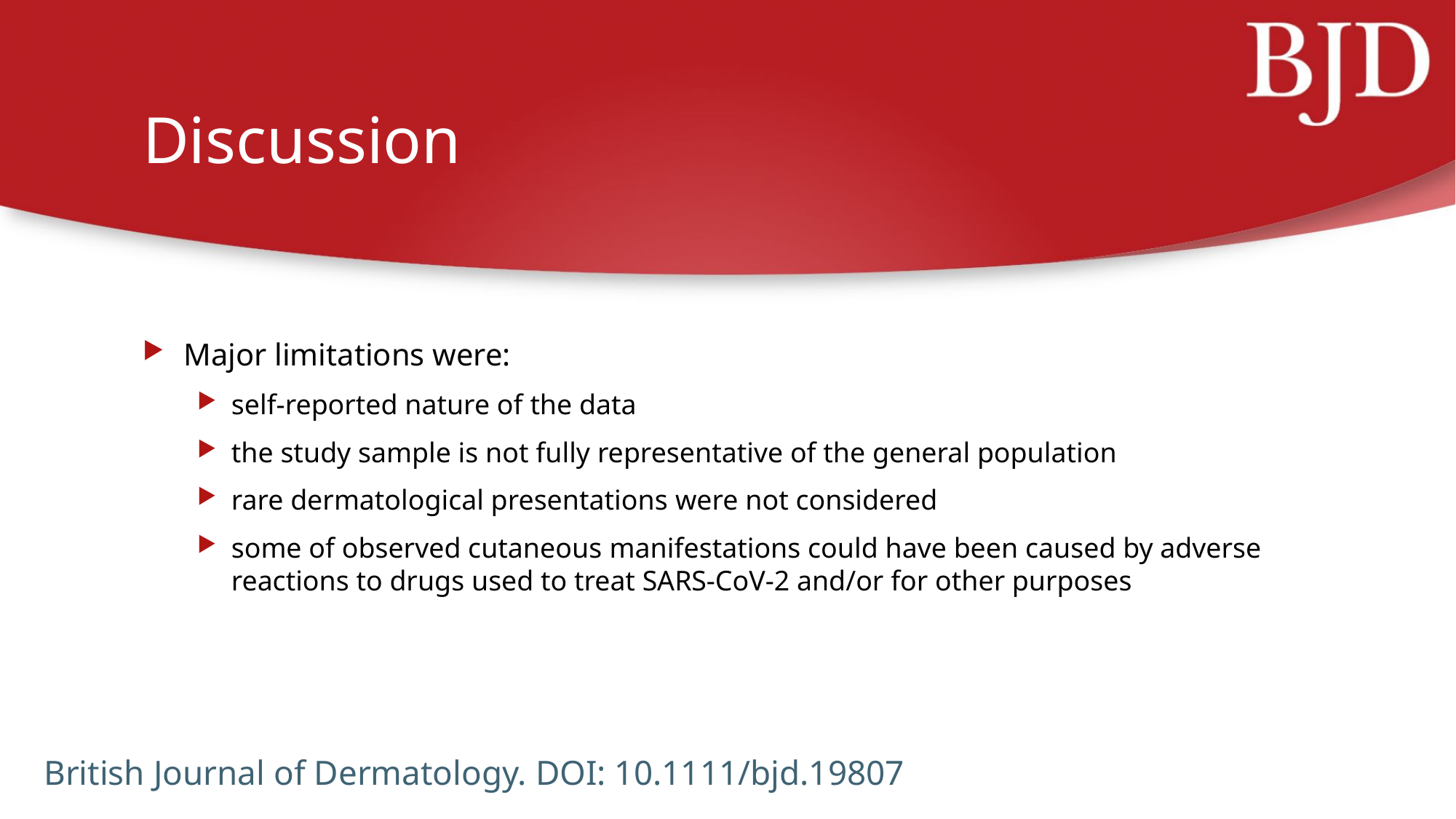

# Discussion
Major limitations were:
self-reported nature of the data
the study sample is not fully representative of the general population
rare dermatological presentations were not considered
some of observed cutaneous manifestations could have been caused by adverse reactions to drugs used to treat SARS-CoV-2 and/or for other purposes
British Journal of Dermatology. DOI: 10.1111/bjd.19807

## Slide 13
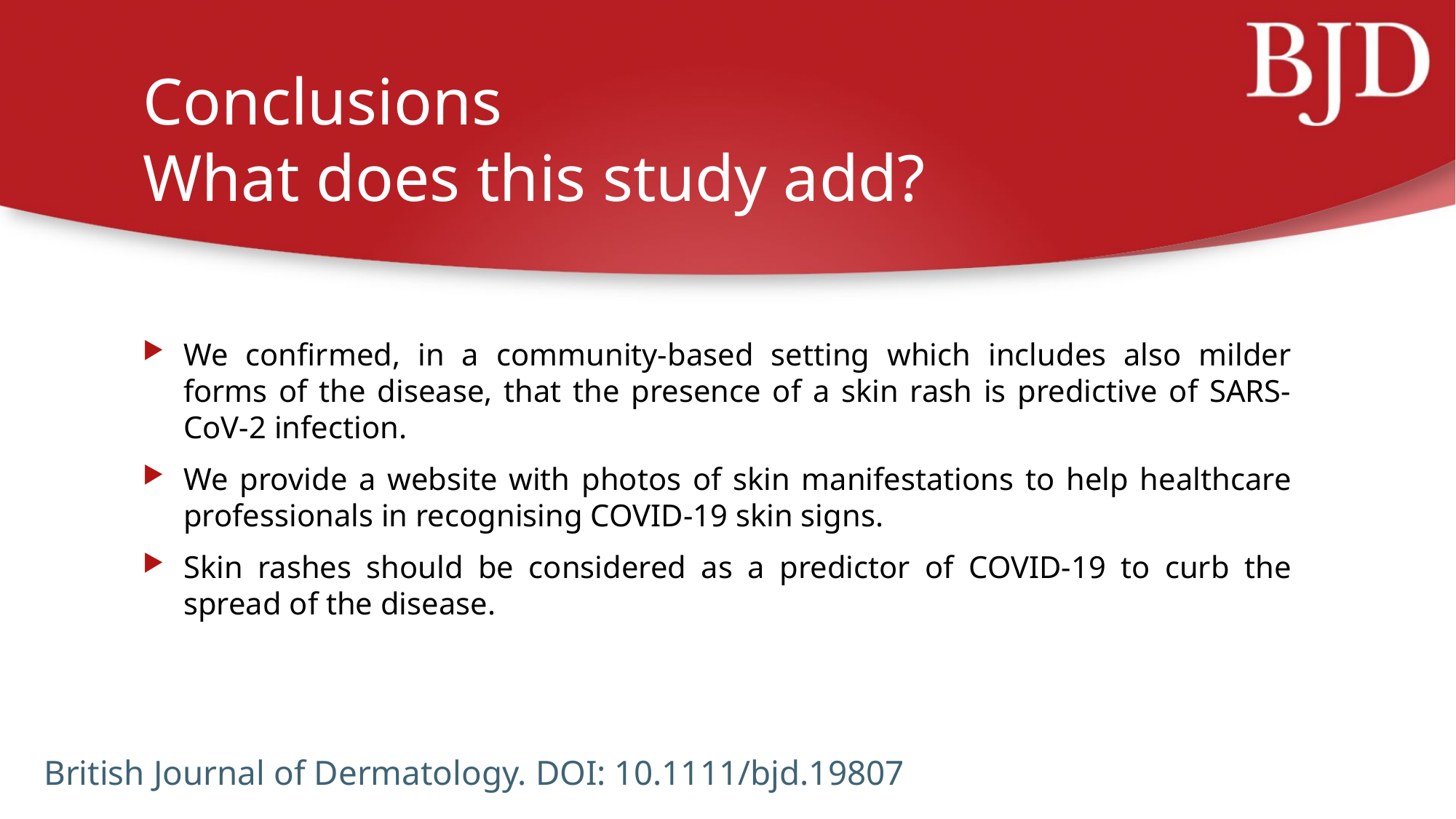

# Conclusions What does this study add?
We confirmed, in a community-based setting which includes also milder forms of the disease, that the presence of a skin rash is predictive of SARS-CoV-2 infection.
We provide a website with photos of skin manifestations to help healthcare professionals in recognising COVID-19 skin signs.
Skin rashes should be considered as a predictor of COVID-19 to curb the spread of the disease.
British Journal of Dermatology. DOI: 10.1111/bjd.19807

## Slide 14
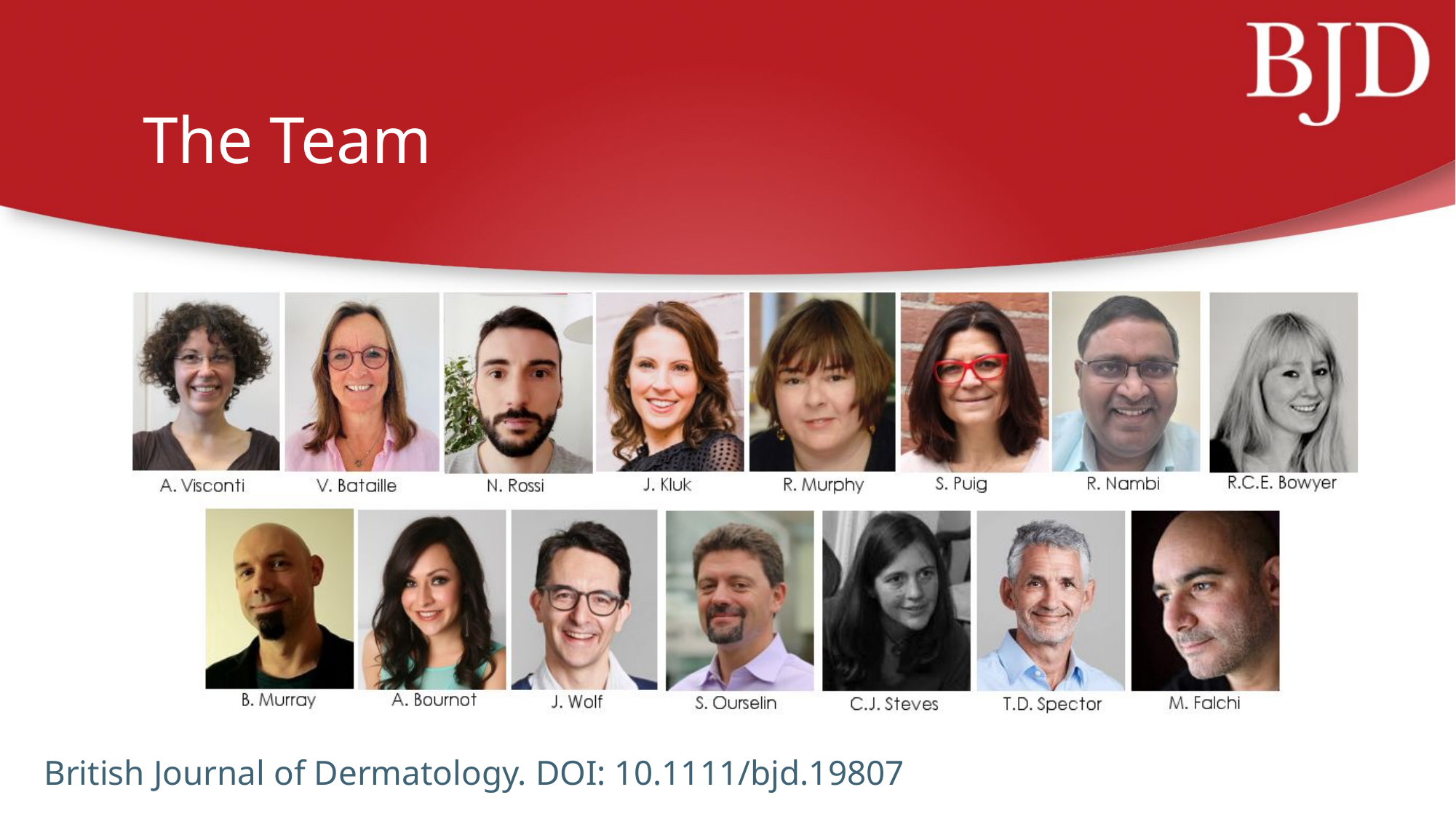

# The Team
British Journal of Dermatology. DOI: 10.1111/bjd.19807

## Slide 15
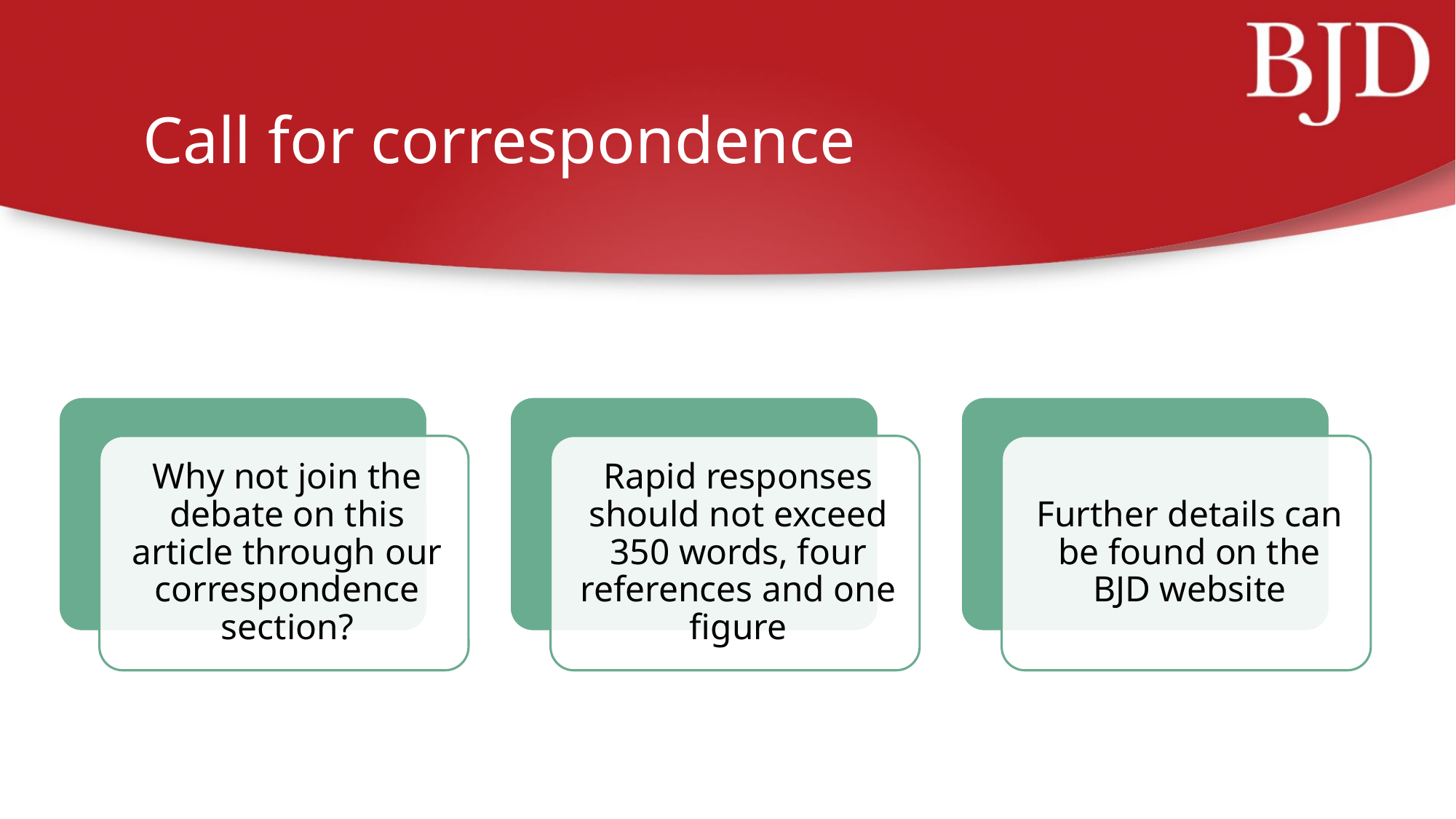

# Call for correspondence
